# Postprandial Metabolic and Oxidative Stress Responses to Grape Pomace Extract in Healthy Normal and Overweight/Obese Women: A Randomized, Double-Blind, Placebo-Controlled Crossover Study

**DOI:** 10.3390/nu15010156

**Published:** 2022-12-29

**Authors:** Maria Choleva, Eleni Matalliotaki, Sokratis Antoniou, Eirini Asimomyti, Archontoula Drouka, Maria Stefani, Mary Yannakoulia, Elizabeth Fragopoulou

**Affiliations:** Department of Nutrition and Dietetics, Harokopio University, 70 Eleftheriou Venizelou Avenue Kallithea, 17671 Athens, Greece

**Keywords:** grape pomace, postprandial, normal weight, overweight/obese, oxidative stress, uric acid, SOD, GPx, TBARS, protein carbonyls

## Abstract

Postprandial oxidative stress has been shown to promote atherosclerosis. Grape pomace (GP) is a source of similar-to-wine bioactive micro-constituents with known antioxidant properties. The aim of the present study was to evaluate metabolic and oxidative stress responses after the intake of grape pomace (GP) extract along with a high-fat meal, in normal and overweight healthy women. In a randomized, double-blind, placebo-controlled crossover study, 18 women were finally included, 11 with BMI < 25 kg/m^2^ and 7 with BMI > 25 kg/m^2^, and consumed a high-fat meal with placebo or GP extract capsules in two separate visits. Blood samples were collected before and 6 h after the consumption. Measurements included basic biochemical markers, uric acid (UA), protein carbonyls (PC), thiobarbituric acid substance (TBARS) levels, as well as superoxide dismutase (SOD) and glutathione peroxidase (GPx) activities. At certain time points, the GP extract consumption in normal-weight women reduced UA, TBARS levels, and SOD activity, whereas it increased UA and reduced PC levels in overweight/obese women, compared to the placebo. GP-derived bioactive compounds may exert antioxidant actions during the postprandial state in healthy women, through different mechanisms according to their BMI status.

## 1. Introduction

It has been proposed that atherosclerosis is related to the postprandial state [1]. The consumption of a high-fat meal has been proposed to promote oxidative stress, which in turn favors endothelial dysfunction and atherogenesis through multiple mechanisms. The typical western dietary patterns lead to repeated exposure to the postprandial elevation of triglycerides, glucose, and insulin, hence to persistent postprandial oxidative stress [2,3]. Excess body weight, which is associated with cardiovascular disease, may contribute to further increases in the oxidative stress response to feeding [4]. Usually, biomarkers that reflect oxidative damage are oxidative products of macromolecules (lipids and proteins) [5], endogenous antioxidant levels, as well as the alteration of antioxidant enzymes’ activity.

Over the years, the scientific literature has provided evidence that phytochemicals derived from fruits and vegetables show potential protective effects against oxidative stress-induced damage [6]. Starting as an epidemiological observation, the possible protective effects of wine consumption on cardiovascular disease mortality were reported in 1979 [7]. Since then, the postprandial and long-term effects of wine ingestion have been widely investigated in randomized controlled clinical studies regarding glucose/insulin and lipid metabolism [8] as well as the redox system [9,10].

The possible cardioprotective effects of wine consumption are attributed mainly to the presence of a mixture of bioactive compounds in suitable proportions [11], rather than its alcohol content [10]. In our previous clinical studies, wine consumption compared to pure alcohol consumption resulted in greater beneficial cardiometabolic outcomes both in healthy men [12,13] and patients with established cardiovascular disease [14,15]. A profitable alternative solution towards the utilization of wine’s beneficial micro-constituents without excess alcohol consumption is the valorization of the vinification by-products, or grape pomace (GP). Although many beneficial micro-constituents are being transferred to wine, especially through the red grape vinification process, wine by-products are still an abundant bioactive compounds source [16]. Additionally, the vinification process produces millions of tons of by-products that also pose an environmental matter of waste management [17].

Several research teams have studied the possible metabolic and antioxidant effects of GP long-term supplementation [16]. Total and LDL cholesterol values were reduced in moderate hyperlipidemic and healthy volunteers receiving GP products compared to a control group [18,19,20], whereas GP-enriched flour reduced insulin levels during an oral glucose tolerance test [21]. Lipid peroxidation products [19,22] and protein carbonyls [22] were also found to be favorably affected by GP supplementation, as well as antioxidant enzymes’ activity [19,23]. However, fewer studies have examined their actions during the postprandial state which entails the elevation of lipids, glucose, and oxidative damage. The consumption of grape pomace or grape seed extract has been shown to reduce postprandial triglycerides levels [24] and glucose levels [25] and improve insulin sensitivity [26]. Moreover, reduced plasma lipid hydroperoxides were reported following a meal supplemented with 300 mg of grape seed extract compared to non-supplemented meal consumption [27]. Taking into consideration that none of these studies have examined the GP supplementation postprandial effect on protein oxidation products and antioxidant enzymes’ activity, a literature gap exists. Additionally, the latter postprandial studies were performed exclusively on male participants; hence, there are no data concerning women’s postprandial response. Considering that estrogens have antioxidant properties, women may be less susceptible to oxidative stress [28].

Therefore, the aim of this study was to evaluate the effect of the acute consumption of a previously characterized GP extract along with a high-fat meal on metabolic and oxidative stress biomarkers in healthy normal-weight and overweight/obese women. The effects of the long-term supplementation of the same GP extract were also investigated in a pilot study with a small sample size.

## 2. Materials and Methods

### 2.1. Study Population

Postprandial study: The participants were apparently healthy women, aged between 22 and 35 years, with a Body Mass Index (BMI) of 20–35 kg/m^2^. Women that received regular prescribed medication or adhered to a weight control diet were excluded from the study. The protocol was approved by the Bioethics Committee of Harokopio University and was in accordance with the Declaration of Helsinki (1989) of the World Medical Association. Potential participants were informed in detail about the study requirements. Initially, 24 women, 14 with BMI < 25 kg/m^2^ and 10 with BMI > 25 kg/m^2^, were recruited according to the inclusion criteria. Finally, 18 of them participated in the study, 11 with BMI < 25 kg/m^2^ and 7 with BMI > 25 kg/m^2^, and gave written consent before participation. The intervention was registered on ClinicalTrials.gov (NCT04436263).

Pilot long-term study: For the pilot, 4 apparently healthy women were recruited, between 28 and 34 years with BMI > 25 kg/m^2^. The same exclusion criteria existed in this pilot study.

### 2.2. Study Design

Postprandial study: The study was a randomized, double-blind, crossover clinical trial. The participants received two different interventions: capsules containing powdered GP extract or placebo capsules, separated by at least a 4-week washout period. Due to the crossover design of the study, volunteers were randomly assigned to a group without knowing which capsules they consumed at the time. Each visit was programmed during the last days of the women’s menstrual cycle in order to limit the potential hormonal effects on the study outcomes. The participants were also advised to limit their usual consumption of foods rich in polyphenols (wine, cocoa, tea, energy drinks, and certain fruits and vegetables) 3 days before the treatment in order to minimize the effect of high polyphenol intake.

On both treatment days, participants came to the Metabolic Unit at the Department of Nutrition and Dietetics of Harokopio University in Athens, Greece after a 10 h fast. A venous catheter was administered and a basal blood sample was obtained (time point −15). Then, within 15 min, the participants consumed a high-fat meal consisting of bread, butter, cheddar, manouri cheese (a traditional Greek cheese with high fat content), salami, and mortadella, along with 6 capsules containing either the GP extract or the placebo. The meal provided 1131 kcal: 66.7% from fats, 19.7% from carbohydrates, and 11.2% from proteins. After the meal’s consumption, blood samples were collected at 0, 30, 60, 90, 120, 150, 180, 210, 240, 300, and 360 min.

Pilot long-term study: A randomized, crossover, double-blind, placebo-controlled long-term pilot study was conducted. The participants were randomly assigned to consume 2 daily capsules of either the GP extract or the placebo for 28 days. At the beginning and at the end of each intervention period, fasting blood samples were collected, on certain days of the menstrual cycle. Both treatments were separated by at least a 4-week washout period. The participants were advised to maintain their usual dietary habits throughout the study.

### 2.3. Grape Pomace Extract

The treatment capsules contained an aqueous-ethanolic red GP extract. The extraction procedure, macronutrient analysis, fatty acids, and phenolic compounds profile were described in a previous publication [29]. A sufficient quantity of the GP was extracted on an industrial scale using the equipment of Cretan Herbal Chem. (www.cretanherbalchem.com) and the capsules’ production was carried out by an appropriate company. The extract was combined with maltodextrin in order to achieve a suitable texture while the placebo capsules contained only maltodextrin. Both capsuled products appeared to be identical in order to maintain the double-blind design of the study. The participants of the postprandial study received an acute dose of 3.5 g of powdered extract (660 mg phenolic compounds as gallic acid equivalents), whereas for the long-term study the participants consumed 1.2 g of powdered extract (220 mg phenolic compounds as gallic acid equivalents) daily for 28 days.

### 2.4. Dietary and Physical Assessment

Postprandial study: In order to evaluate participants’ compliance in the washout period, as well as the differences in dietary consumption between the two visits, three 24 h recalls were collected prior to each treatment. Energy, macro-, and micronutrient intake were analyzed using the Nutritionist Pro version 2.2 software (Axxya Systems-Nutritionist Pro, Stafford, TX, USA). A validated food frequency questionnaire (FFQ) [30] was filled out in both visits in order to assess the last month’s dietary intake and was used to assess the level of adherence to the Mediterranean diet through the MedDietScore [31]. Physical activity was assessed with the use of a validated questionnaire (IPAQ-short version) [32] and Physical Activity Level (PAL) was calculated for each volunteer.

Pilot long-term study: At the beginning and at the end of each intervention period, participants completed an FFQ and an IPAQ-short version (4 of each questionnaire in total) in order to estimate the differences in dietary intake and physical activity before and throughout the study. In order to enhance participants’ compliance with the study requirements, a reminder call was made each week; they were also asked to bring the used capsules back to the research team.

### 2.5. Anthropometry and Blood Sample Collection

For both studies, anthropometric measurements were made on each visit. Weight was measured to the nearest 0.1 kg using a digital scale and height to the nearest 0.1 cm using a stadiometer (in light clothing and without shoes). The body fat (%), body fat (kg), and body fat-free mass (kg) of each participant were measured with bioelectrical impedance analysis (Tanita BC-418 MA, Tanita Corp., Tokyo, Japan). Waist circumference was measured to the nearest 0.1 cm between the superior iliac crest and the lower rib margin in the mid-axillary line. Resting arterial blood pressure was measured in the right arm with the participant in a sitting position. 

Venous blood samples were drawn and serum was isolated after a 45-min incubation at room temperature by centrifugation at 1500× *g* for 10 min. Plasma was isolated in EDTA vacutainers after immediate centrifugation at 1500× *g* for 10 min. For the isolation of leukocytes, 4 mL of heparinized blood was obtained and leukocytes were isolated, as previously described [33]. The protein content of all preparations was determined according to the Bradford method [34], with the use of bovine serum albumin as a protein standard. All biological samples were immediately aliquoted and stored at approximately −80 °C.

### 2.6. Basic Biochemical Markers

Glucose levels (glucose oxidase, sensitivity 0.7 mg/dL, intra-assay coefficient of variation (CV) 2.4%), total cholesterol (TC) (cholesterol esterase/cholesterol oxidase, sensitivity 4 mg/dL, intra-assay CV 1.6%), high-density lipoprotein (HDL) (cholesterol was determined using the same procedure, after the precipitation of non-HDL lipoproteins with phosphotungstic acid), and uric acid (UA) (uric acid uricase, sensitivity 0.2 mg/dL, intra-assay CV 1.1%) were measured in serum samples using colorimetric kits (Biosis, Biotechnological Applications Ltd., Athens, Greece). Low-density lipoprotein (LDL) cholesterol levels were calculated using the Friedewald formula. Triglycerides (TG) were measured with the Atellica CH 930 Analyzer (Siemens Healthcare Diagnostics Inc., New York, NY, USA) (sensitivity 4 mg/dL, intra-assay CV 2.5%), and insulin levels were determined with the Atellica IM 1600 Analyzer (Siemens) (sensitivity 0.8 μIU/mL, intra-assay CV 3.1%).

### 2.7. Thiobarbituric Acid Reactive Substances (TBARS)

TBARS levels were measured in serum using a modified colorimetric method [35]. The assay mixture contained 0.1 mL of serum, 0.2 mL of phosphoric acid 0.2 Μ, 0.025 mL of butylated hydroxyl toluene (BHT) 5 mM, and 0.025 mL of thiobarbituric acid 0.11 M. The mixture was incubated for 60 min at 90 °C and then cooled, and after the addition of 0.5 mL butanol, it was centrifuged (12,000× *g*, 10 min). The butanolic phase was collected, centrifuged (12,000× *g*, 10 min), and transferred into a 96-well plate, and the absorbance was measured at 532 nm. The analysis was conducted with the use of a microplate spectrophotometer (BioTek PowerWave XS2, Agilent Technologies, Highland Park, IL, USA). TBARS concentration was calculated using 1, 1, 3, 3-tetramethoxypropane as a standard. Results are expressed as μM.

### 2.8. Protein Carbonyls

The Bradford method [34] was initially used for the estimation of the protein content in serum samples. A 0.1 mL measure of 2, 4-dinitrophenylhydrazine (DNPH) 10 mM was added into serum containing 1 mg of protein. Samples were incubated for 10 min at room temperature, and then a 0.03 mL 100% trichloroacetic acid (TCA) solution was added. After 5 min of incubation in ice, the samples were centrifuged (13,000× *g*, 2 min) and the supernatant was discarded. A 0.5 mL measure of cold acetone was then added to the sediments, and after a 30 s sonication process, they were incubated at −20 °C for 5 min. Samples were centrifuged (13,000× *g*, 10 min) in order to remove the supernatant and the sediments were dissolved in 0.2 mL guanidine hydrochloride 6 M. A 0.1 mL measure of each sample was transferred into a 96-well plate, and absorbance was measured at 375 nm. In order to estimate the remaining protein content in the samples, absorbance was read at 280 nm. The analysis was conducted with the use of a microplate spectrophotometer (BioTek PowerWave XS2). Results are expressed as μmol protein carbonyls per μg of protein.

### 2.9. Superoxide Dismutase (SOD) Activity

Total SOD activity was measured in leukocyte-rich plasma (LRP) according to the method of McCord et al. [36]. The sample volume was adjusted until the inhibition of SOD was achieved between 40 and 60%, representing the linear part of the inhibition curve. SOD from bovine erythrocytes was used to calculate the standard curve. The analysis was conducted with the use of a spectrophotometer (Novaspec II, Pharmacia Biotech, Cambridge, England). Units were calculated based on the formula SOD Units = %inhibition/(100%inhibition), and the results are expressed as units per mg of protein.

### 2.10. Glutathione Peroxidase (GPx) Activity

The GPx activity was measured in plasma by the continuous monitoring of the regeneration of reduced glutathione (GSH) from oxidized glutathione (GSSG) upon the action of glutathione reductase (GR) and NADPH [37]. The analysis was conducted with the use of a microplate spectrophotometer (BioTek PowerWave XS2). Results are expressed as units of GPx per L of serum.

### 2.11. Statistics

Power analysis, based on our previous postprandial study (published and unpublished data) [12], showed that a total number of 20 participants, or 10 participants using a crossover study design, was adequate to achieve a statistical power of >80% at a 5% significance level with an effect size of 0.23 (the minimum of glucose, insulin, UA, TBARS) for the comparison of two groups and using 7 time measurements (a range of 7–12 time points was measured in the present study). Power analysis for the comparison of four groups showed that a total number of 32 participants, or 16 participants using a crossover study design, was adequate to achieve a statistical power of >80% using 7 time measurements.

The normality of the variables was tested using the Kolmogorov–Smirnov test. Normally distributed variables are presented as mean values ± SD (standard deviation), while skewed variables are presented as medians (25th–75th quartile). The Independent Samples *t*-test or Mann–Whitney U tests were used for the comparisons of baseline values according to their distribution. During the intervention, repeated measures ANOVA was used for the comparisons (p_time_, p_trial_, p_time*trial_) of normally distributed variables. The paired samples *t*-test was used to compare each time point to the baseline and between interventions. For skewed variables, Friedman’s 2-way ANOVA by ranks was used for the estimation of the time effect (p_time_) in each intervention group and the Wilcoxon paired test was used for the differences at each time point compared to the baseline. Trial-related pairwise comparisons were performed using the Wilcoxon paired test. Net incremental areas under the curve (iAUC) were calculated using the GraphPad Prism 8 software. The associations between variables were analyzed using the Pearson correlation. Analyses were conducted for the total sample population as well as with respect to the BMI group. Missing values (<1.5%) were calculated using the mean of the percentage of change at each time point in the same intervention and BMI group. All reported *p*-values are compared to a significance level of 5%. The STATA Statistical Software, Release 12 (STATA Corp., College Station, TX, USA), was used for statistical analyses.

## 3. Results

### 3.1. Baseline Anthropometric, Biochemical, and Oxidative Stress Markers

Initially, 24 women were recruited, and eventually, 18 of them participated in the postprandial study. Out of the 18 participants, 11 of them had BMI values < 25 kg/m^2^, and the other seven had BMI > 25 kg/m^2^. No differences were observed in baseline anthropometric and biochemical markers between the two interventions in both groups, BMI < 25 kg/m^2^ and BMI > 25 kg/m^2^ (Table 1). As expected, significant differences were found between BMI groups concerning body weight, BMI, waist circumference, hip circumference, body fat (kg, %), and body fat-free mass. Basic biochemical markers did not exceed the normal range in any BMI group at baseline, indicating a healthy study population.

The baseline values of uric acid (UA), TBARS, protein carbonyls (PC), SOD activity, and GPx activity did not differ significantly between interventions, nor for the whole study sample (data not shown), nor for each BMI group (Table 2).

### 3.2. Dietary Assessment and Study Compliance

MedDietScore prior to each intervention did not differ between the two groups (placebo: 34.4 ± 4.0, extract: 34.6 ± 4.5; *p* = 0.88). Similar results were separately obtained in the analysis of the BMI < 25 kg/m^2^ group (*p* = 0.67) and the BMI > 25 kg/m^2^ group (*p* = 0.38).

According to the data from 24 h dietary recalls, no significant differences were observed regarding energy intake, the consumption of macronutrients, vitamins, basic food groups, and physical activity between the washout periods of the two interventions (data not shown). Moreover, the sub-analysis by BMI did not show any differences prior to the interventions (Table 3).

In order to assess participants’ compliance with the study requirements, the last month’s daily consumption (FFQ data) of fruits and vegetables was compared to the consumption 3 days before the interventions (24 h recall data). Indeed, participants before the placebo intervention limited their consumption of fruits and vegetables by 2.3 servings per day (*p* < 0.01) and before the extract intervention by 1.9 servings per day (*p* < 0.01). Participants also limited their wine consumption prior to the placebo intervention by 0.14 servings per day (*p* = 0.04) and prior to the extract intervention by 0.07 servings per day (*p* < 0.01).

### 3.3. Basic Biochemical Markers during Interventions

The basic biochemical marker responses are presented in Table 4 for the whole study sample and in Supplementary Tables S1 and S2 for BMI groups < 25 kg/m^2^ and >25 kg/m^2^, respectively.

Concerning glucose levels, no significant overall changes were observed between interventions in the total participants, except for a tendency for a time effect (Table 4). During the placebo intervention, glucose levels increased significantly at 30 min and decreased at 60 min compared to baseline values (Table 4). The consumption of the extract along with the meal did not change the glucose response (Table 4). Subgroup analysis with respect to BMI revealed a significant time effect in participants with BMI < 25 kg/m^2^ (p_time_ = 0.04, Table S1). In the extract intervention, both BMI groups showed increased glucose at 30 min (by 10.9%, p_BMI < 25_ = 0.02, Table S1 and by 15.9%, p_BMI > 25_ = 0.04, Table S2). Moreover, participants with BMI > 25 kg/m^2^ presented initially higher values (p_0_ = 0.03) and later lower values (p_240_ = 0.03, p_300_ = 0.01, p_360_ = 0.04) compared to the BMI < 25 kg/m^2^ group (Tables S1 and S2).

Compared to baseline values, insulin levels escalated significantly between 30 and 150 min in both interventions, for total participants (Table 4), as well as for the individual BMI groups (Tables S1 and S2). The time effect was significant for total participants (p_time_ < 0.01) and for each BMI group (p_timeBMI < 25_ < 0.01, p_timeBMI > 25_ = 0.03); however, no trial effect was found. The comparison of the two interventions showed that insulin levels at 60 min were markedly increased for the extract compared to the placebo intervention by 135.2% (p_P-E_ = 0.04, Table 4), which was mainly attributed to participants with BMI < 25 kg/m^2^ (96.5%, p_P-E_ = 0.05, Table S1).

A significant time effect was revealed for TG levels in total participants (p_time_ < 0.01, Table 4). Both in the placebo and extract interventions, the consumption of the high-fat meal promoted TG elevation from 30 min until the end of the 6 h study compared to baseline values (Table 4). Although the trial effect was not significant, the extract consumption led to lower TG values compared to the placebo intervention at 300 min by 31.4% (p_P-E300_ = 0.04, Table 4). Subgroup analysis revealed a significant time effect only in participants with BMI < 25 kg/m^2^ (p_time_ < 0.01, Table S1); however, both BMI groups showed increased TG levels from 30 min until the end of the 6 h study compared to baseline values (Tables S1 and S2). In the extract intervention, TG elevation was significantly lower for participants with BMI > 25 kg/m^2^ compared to participants with BMI < 25 kg/m^2^ at 360 min (p_360_ = 0.002) (Tables S1 and S2). Concerning the other lipid biomarkers, a significant time effect was only observed concerning HDL cholesterol and LDL cholesterol (Table 4). Subgroup analysis, based on BMI, revealed that this time effect is presented in the BMI < 25 kg/m^2^ group (Tables S1 and S2).

### 3.4. Oxidative Stress Markers during Interventions

**Figure 1:**
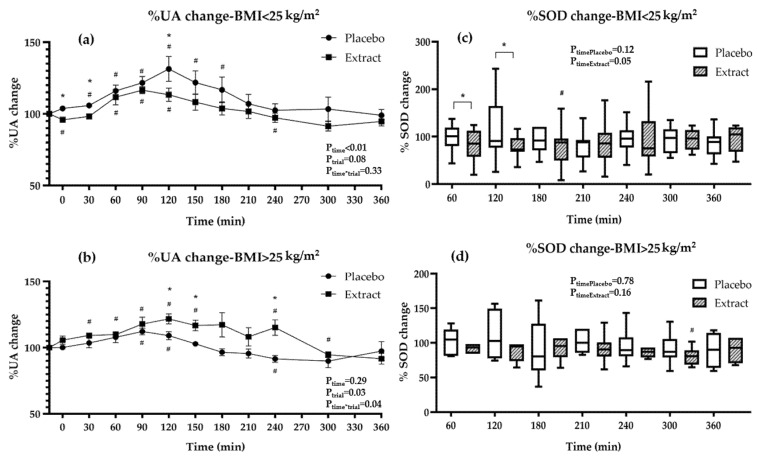
Percentage of change in endogenous antioxidant biomarkers between interventions for (**a**,**c**) participants with BMI < 25 kg/m^2^, (**b**,**d**) participants with BMI > 25 kg/m^2^. Data are presented as means ± SEM or median (25th–75th quartile). BMI, body mass index; SOD, superoxide dismutase; UA, uric acid. P_time_, P_trial_, P_time*trial_ from RMANOVA, P_timePlacebo/Extract_ from Friedman’s 2-way ANOVA. ^#^ *p* ≤ 0.05 compared to baseline values from paired samples *t*-test or Wilcoxon paired test, respectively. * *p* ≤ 0.05 between interventions from paired samples *t*-test or Wilcoxon paired test, respectively.

**Figure 2:**
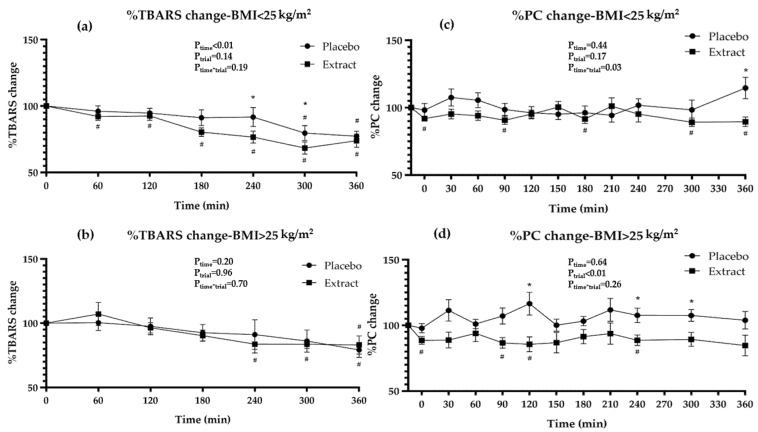
Percentage of change in oxidative biomarkers between interventions for (**a**,**c**) participants with BMI < 25 kg/m^2^, (**b**,**d**) participants with BMI > 25 kg/m^2^. Data are presented as means ± SEM. BMI, body mass index; TBARS, thiobarbituric acid reactive substances; PC, protein carbonyls. P_time_, P_trial_, P_time*trial_ from RMANOVA. ^#^ *p* ≤ 0.05 compared to baseline values from paired samples *t*-test, * *p* ≤ 0.05 between interventions from Paired samples *t*-test.

The potential antioxidant actions of the GP extract consumption along with a meal were evaluated by measuring endogenous antioxidants (UA), oxidative products derived from macromolecular damage (TBARS, PC), and antioxidant enzyme activity (SOD and GPx). The postprandial responses are presented in Table 5, in Figure 1 and Figure 2, and in Tables S3 and S4.

Concerning UA levels, a significant time effect (p_time_ < 0.01), but no significant trial or trial x time effect, was observed (Table 5). When sub-group analysis was performed with respect to BMI (Figure 1a,b; Tables S3 and S4), participants with BMI < 25 kg/m^2^ revealed a significant time effect (p_time_ < 0.01) and a trend for a trial effect (p_trial_ = 0.08) (Table S3), and participants with BMI > 25 kg/m^2^ showed a significant trial effect (p_trial_ = 0.03) and trial x time effect (p_time*trial_ = 0.04) (Table S4). These results indicate different responses in uric acid postprandial modulation in the two BMI groups. In the placebo intervention, the consumption of the high-fat meal in participants with BMI < 25 kg/m^2^ revealed an increase at 30–150 min, while those with BMI > 25 kg/m^2^ increased in a smaller time range (90–120 min) and to a lesser extent (BMI < 25 kg/m^2^ vs. BMI > 25 kg/m^2^: p_120_ = 0.02, p_180_ = 0.05, p_240_ < 0.01). In the extract intervention, the consumption of the high-fat meal, in participants with BMI < 25 kg/m^2^, mainly increased UA levels at 60–120 min, while participants with BMI > 25 kg/m^2^ revealed elevated UA levels for a longer period (30–150 min and 240 min) and to a greater extent (BMI < 25 kg/m^2^ vs. BMI > 25 kg/m^2^: p_0_ < 0.01, p_30_ < 0.01, p_240_ = 0.01). Both groups showed reduced UA levels at 300 min compared to baseline (Figure 1a,b; Tables S3 and S4). The comparison of the two interventions in participants with BMI < 25 kg/m^2^ indicated elevated UA levels in the placebo compared to the extract intervention at several time points (p_P-E0_ = 0.01 by 9%; p_P-E30_ = 0.01 by 7.7%, p_P-E120_ = 0.02 by 18%) (Figure 1a, Table S3). Concerning participants with BMI > 25 kg/m^2^, UA levels appeared higher in the extract compared to the placebo intervention at several time points (p_P-E120_ = 0.02 by 12.6%; p_P-E150_ = 0.01 by 13.9%; p_P-E240_ < 0.00 by 23.7%) (Figure 1b, Table S4).

Postprandial TBARS responses revealed a significant time effect (p_time_ < 0.01, Table 5) for total participants. When subgroup analysis was performed with respect to BMI (Figure 2a,b; Tables S3 and S4), only participants with BMI < 25 kg/m^2^ revealed a significant time effect (p_time_ < 0.01), indicating different responses in the two BMI groups. Surprisingly, the high-fat meal, during the placebo intervention, revealed reduced lipid peroxidation (Table 5). Specifically, participants with BMI < 25 kg/m^2^ showed decreased TBARS levels at 300 and 360 min (p_300_ < 0.01, p_360_ < 0.01) (Figure 2a, Table S3), whereas those with BMI > 25 kg/m^2^ showed decreased values only at 360 min (*p* = 0.01) (Figure 2b, Table S4) compared to baseline. No differences were observed between the two BMI groups. In the extract intervention, TBARS levels were decreased for a more extended time period (Table 5). Especially in the BMI < 25 kg/m^2^ group, the extract consumption along with the meal reduced TBARS levels at all measured time points (Figure 2a, Table S3), whereas in the BMI > 25 kg/m^2^ group, TBARS levels were reduced after 240 min (Figure 2b, Table S4) compared to baseline levels. The comparison between the two interventions revealed that only in the BMI < 25 kg/m^2^ group did the extract intervention tend to decrease TBARS levels at 180 min by 10.8% (p_P-E180_ = 0.06) and significantly decreased TBARS by 15.1% and 11.2% at the next two time points (p_P-E240_ = 0.02, p_P-E300_ = 0.01) compared to the placebo intervention (Figure 2a, Table S3).

Concerning PC levels, significant trial effect (p_trial_ < 0.01) and trial x time interaction (p_time*trial_ = 0.03) were found for total participants. In the placebo intervention, the consumption of the high-fat meal did not affect PC levels in the total sample (Table 5). On the other hand, during the extract intervention, PC levels were decreased at several time points, by a range of 7.2–12.4%, compared to baseline (Table 5). The comparison of the two interventions revealed significantly lower PC levels (ranging between 10.6% and 22.8%) in the extract compared to the placebo intervention at several time points (Table 5). The subgroup analysis with respect to BMI showed that a significant trial x time interaction was observed (p_time*trial_ = 0.03) in the BMI < 25 kg/m^2^ group, and a trial effect was observed (p_trial_ < 0.01) in the BMI > 25 kg/m^2^ group. In the placebo intervention, the consumption of the high-fat meal did not affect PC levels in any of the BMI groups (Figure 2c,d; Tables S3 and S4) compared to baseline levels. However, the BMI > 25 kg/m^2^ group presented increased PC levels (p_120_ = 0.04, p_210_ = 0.05) compared to the BMI < 25 kg/m^2^ group (Tables S3 and S4). In the extract intervention, both BMI groups revealed significant reductions at several time points compared to baseline (Figure 2c,d; Tables S3 and S4). In participants with BMI < 25 kg/m^2^, the reduction ranged between 8.1 and 10.8%, whereas in the BMI > 25 kg/m^2^ group, the reductions ranged between 11.4 and 14.5%. The comparison of the two interventions showed that participants with BMI > 25 kg/m^2^ significantly decreased PC values at more time points than participants with BMI < 25 kg/m^2^ compared to placebo (Figure 2c,d; Tables S3 and S4).

The placebo intervention did not affect SOD activity; however, a significant time effect was revealed in the extract intervention for total participants (p_time_ = 0.04, Table 5). Specifically, in the extract intervention, SOD activity decreased at several time points compared to baseline (Table 5). The comparison between the two interventions showed that participants during the extract intervention displayed decreased SOD activity at 60 min by 13.6% and at 120 min by 22.4% compared to the placebo intervention (Table 5). Subgroup analysis showed that the above changes were detected in the participants with BMI < 25 kg/m^2^ (p_time_ = 0.05, Figure 1c, Table S3). Specifically, the extract consumption along with the meal resulted in more pronounced reductions in SOD activity compared to baseline in the BMI < 25 kg/m^2^ group than in BMI > 25 kg/m^2^ group (Figure 1c,d; Tables S3 and S4). The comparison of the two interventions, in each BMI group, showed that only in participants with BMI < 25 kg/m^2^ did the extract intervention significantly reduce SOD activity at 60 min by 15.7% (p_P-E_ = 0.04) and at 120 min by 18.2% (p_P-E_ = 0.04) compared to the placebo (Figure 1c,d; Tables S3 and S4). GPx activity was not affected overall during both interventions for total participants) (Table 5) or for any of the BMI groups (Tables S3 and S4). GPx activity was slightly increased immediately after meal consumption by 3.7% and 4.6% in the placebo and extract intervention, respectively, compared to baseline (Table 5).

### 3.5. Net iAUCs

Net iAUCs were calculated in order to further assess the metabolic and oxidative stress-related responses in both interventions in the specific participants. Correlations of oxidative stress net iAUC values with basic biochemical markers were investigated. The consumption of the meal, placebo intervention, revealed a significant positive correlation between the iAUCs of glucose and SOD (r = 0.463, *p* = 0.05), of TC and UA (r = 0.657, *p* = 0.003), of TC and GPx (r = 0.711, *p* = 0.001), of HDL and GPx (r = 0.614, *p* = 0.007), and of UA and GPx (r = 0.717, *p* = 0.001). Moreover, a negative correlation was observed between LDL and SOD (r = −0.461, *p* = 0.05) and SOD and PC (r = −0.449, *p* = 0.06). The subgroup analysis based on BMI revealed that in BMI < 25 kg/m^2^, the correlations of glucose/SOD, TC/UA, TC/GPx, and HDL/GPx were retained, while in BMI > 25 kg/m^2^, the correlation of UA/GPx was retained. Moreover, some correlations were found only in the BMI < 25 kg/m^2^ group (glucose/PC, r = −0.627, *p* = 0.003) and only in the BMI > 25 kg/m^2^ group (glucose/TG, r = 0.780, *p* = 0.03; insulin/PC, r = 0.674, *p* = 0.09; HDL/SOD, r = 0.867, *p* = 0.01).

Postprandial PC net iAUC was significantly lower for total participants and participants with BMI > 25 kg/m^2^ after the extract compared to the placebo intervention (*p* < 0.01). Participants with BMI > 25 kg/m^2^ tended to have increased UA net iAUC after the extract compared to the placebo intervention (*p* = 0.08). In the placebo intervention, the UA net iAUC of participants with BMI < 25 kg/m^2^ tended to be higher compared to participants with BMI > 25 kg/m^2^ (*p* = 0.07), whereas in the extract intervention, the BMI < 25 kg/m^2^ group tended to have lower net iAUC compared to the BMI > 25 kg/m^2^ group (*p* = 0.07).

### 3.6. Pilot Long-Term Study

The participants of the pilot study consumed 93% of the placebo capsules and 90% of the extract capsules. Basic biochemical or oxidative stress markers did not differ at baseline before each intervention period (data not shown). No changes were detected in interventions compared to their baseline values, except for TC and PC, which tended to decrease after the extract intervention by 10.2% and 12.4%, respectively (*p* = 0.06, Table 6). No differences between interventions were reported.

## 4. Discussion

The consumption of GP extract along with a meal reduced UA levels, SOD activity, and TBARS levels in normal-weight participants, whereas it increased UA levels and decreased PC levels in overweight/obese participants, compared to the placebo intervention. To our knowledge, this is the first study to evaluate the metabolic and antioxidant responses of a GP extract along with the consumption of a standardized high-fat meal in healthy women with respect to normal-weight and overweight/obese status.

The consumption of a high-caloric meal consisting of saturated fats and sugars promotes the elevation of postprandial plasma TG and glucose, which contribute to metabolic and signaling dysregulations, including oxidative stress [38]. Sex could also influence postprandial metabolism, since women are more susceptible to the inhibitory effect of insulin on endogenous glucose production and present more efficient peripheral TG clearance and lower levels of plasma TG after meal consumption than men, differences probably attributed to the presence of sex hormones [39].

In the present study, only women participated, and the meal provided 1131 kcal (66.7% fat—saturated fatty acids, SFA, were approximately 45% of total fat—19.7% carbohydrates, 11.2% protein), similar to other standardized high-fat meals reported [40,41]. Following the meal’s ingestion (placebo intervention), glucose and insulin levels peaked at 30 min and TG levels were elevated from 30 min throughout the 6 h intervention, with peak values detected between 120 and 150 min, according to the literature [2]. Theoretically, the logical hypothesis is that the postprandial period leads to increased oxidative stress. Both dietary carbohydrates and fats were capable of producing an increase in oxidation products, and when fed together, they may have an additive effect. Concerning lipid peroxidation, oxidized lipids could either have a dietary origin or be endogenously produced. The majority of studies support an increase in lipid peroxidation measured either as TBARS or MDA levels after the consumption of a mixed meal or after an oral glucose test, mainly in diabetic patients, and a weaker increase in healthy volunteers [42]. However, there are studies that did not detect an increase in TBARS levels after carbohydrate [43,44,45,46,47,48] or fat [49,50] consumption in healthy volunteers. Indeed, in one study, lipid peroxidation decreased after fat ingestion, even though the change was small [49]. Similarly, to our surprise, in the present study, TBARS values were decreased at 300–360 min after meal consumption. Differences between the age of the participants, the amount of calories given, the presence or absence of obesity, and the lipid composition of the meals may be some reasons for the discrepancies between the studies. The lipid composition may also influence lipid peroxidation since SFA 40–80% of total fat has usually [51,52,53,54,55], but not always [44,50,56], led to increased TBARS or MDA levels. Overweight and mainly obesity could influence oxidative stress status. Our overweight/obese female volunteers did not reveal higher oxidative stress markers compared to the normal-weight ones at baseline. This is in agreement with previous studies that suggested a sex dependence in the relationship of obesity with oxidative damage [49,57]. However, normal-weight females revealed greater identified TBARS reductions compared to the overweight/obese ones after meal consumption. Limited data exist, in particular only for a healthy population, concerning the levels of protein oxidation products after meal consumption. Two studies showed an increase in advanced oxidative protein products [58] and in PC [59] after the consumption of a fat meal, while one revealed no change in PC levels [60]. In the present study, the consumption of the high-fat meal did not affect PC levels, either in total participants or in any of the BMI groups. On the other hand, UA levels increased significantly after meal consumption between 30 and 150 min, and a more pronounced effect was observed in normal-weight participants. Indeed, the iAUCs of UA tended to differ between the two BMI groups after the high-fat meal consumption. UA is capable of scavenging reactive oxygen species and chelating iron and copper ions, hence inhibiting reactions such as Fenton or Haber–Weiss and modulating the activities of antioxidant enzymes [61]. UA postprandial levels could be attributed either to dietary intake through the meal or to endogenous production. The meal in the present study had approximately 66 mg of purines [62], which could contribute to a lesser extent to the postprandial increase. On the other hand, it has already been proposed that UA levels rise after the consumption of a high-fat meal as an endogenous antioxidant mechanism [63]. Finally, it should be noticed that despite a slight increase, and maybe without physiological significance, in GPx activity immediately after the meal consumption, the two enzymes, SOD and GPx, were not significantly affected during the placebo intervention. It is possible that the rise in UA levels in this study may have prevented the elevation of lipid and protein oxidation biomarkers. It should also be noticed that although the enzymes’ activity was not significantly changed during the high-fat meal consumption, a positive correlation was found between the iAUCs of glucose and SOD, TC/HDL and GPx, and UA levels and GPx, and a negative correlation was found between GPx activity and PC levels in total participants. Especially in the normal-weight women, glucose was positively correlated with SOD activity and SOD activity negatively correlated with PC levels, whereas in overweight/obese women, a trend for a correlation between insulin and PC was observed as well as a positive correlation between HDL and SOD.

The detailed chemical composition of the extract, from the red grape varieties Cabernet Sauvignon, Cabernet Franc, Syrah, and Sour Black, has been previously published, as well as its in vitro biological actions. Among its bioactive compounds are phenolic compounds (9.64 ± 1.29 g gallic acid equivalents per 100 g) and phospholipids (7.07 ± 1.02 g phosphorus per 100 g of extract). The most abundant phenolic compounds identified were catechin, epicatechin, quercetin, and malvidin-3-*O*-glucoside, and from fatty acids, linoleic acid [29]. The specific extract exerts, in vitro, potent antioxidant effects, since it successfully scavenges DPPH radicals and delays Cu-induced serum oxidation. Therefore, the aim of the present work was to investigate the possible in vivo antioxidant effects of the specific extract, taking into consideration the very important process of the absorption and bioavailability of its bioactive compounds. For this purpose, the women in the study consumed an acute dose of 3.5 g of powdered extract (660 mg phenolic compounds as gallic acid equivalents) along with the high-fat meal.

After the extract consumption, along with the meal, no differences were shown concerning the glucose and insulin response compared to the placebo intervention for total participants or any of the BMI groups. The only exception was that insulin levels at 60 min were significantly increased by 96.5% in the extract compared to the placebo intervention in normal-weight participants. Limited data exist in the literature and no similar clinical trial exists in order to directly compare the results of the present study. However, in metabolic syndrome volunteers, both men and women, the consumption of grape seed along with a meal (670 kcal, 30% fat) reduced the postprandial glucose response, but not that of insulin [64]. In contrast, the consumption of a red GP drink, along with a meal (970 kcal, 40% fat), reduced postprandial insulin levels and improved insulin sensitivity in healthy men [26]. Moreover, the intake of grape seed extract with a high-carbohydrate meal (73.6% carbohydrates) reduced postprandial glucose levels at 15 and 30 min in healthy volunteers [25]. It is possible that the controversies of the studies may be due to differences in the sex of the participants and/or the composition of the meals or the composition of the extract. Concerning TG levels, no overall trial effect was observed, but significantly decreased values after the extract consumption compared to the placebo intervention were reported at 300 min by 45% in normal-weight women. In accordance, the postprandial modulation of TG levels by grape products was not shown in other studies [26,27,64]. It is possible that long-term supplementation is needed in order to effectively reduce postprandial [24] or baseline TG levels [18].

Concerning UA, during the extract intervention, UA levels were increased between 60 and 150 min in total participants. Interestingly, the response of the two BMI groups was different when compared to the placebo treatment. Specifically, in the normal-weight women, UA levels were lower at several time points in the extract intervention compared to the placebo one. In contrast, in the overweight/obese women, the levels were higher at several time points in the extract intervention compared to the placebo one. These findings support a BMI-dependent effect of the extract on postprandial UA levels. Another study that provided a high-fat meal with an antioxidant beverage that contained, among other things, grape seed and skin extracts, showed that UA levels did not increase as much in the beverage meal as in the control meal intervention [63], as we found in the normal-weight women. This is possibly due either to the preferential utilization of the exogenous antioxidants (leading indirectly to the inhibition of UA production) and/or due to the direct polyphenol-induced inhibition of liver xanthine oxidoreductase. However, it is reported that the phenolic compounds might act via other mechanisms apart from the simple inhibition of enzyme activities since the decrease in UA levels did not correlate with xanthine dehydrogenase and xanthine oxidase activities in experimental animals [65]. The difference between the two BMI groups may lie in the different xenobiotic transport and metabolism that are dependent on normal metabolic regulation. It is possible that in overweight/obese women these systems are altered and, by extension, affect the bioavailability and effectiveness of phenolic compounds.

The extract consumption along with the high-fat meal resulted in a reduction in lipid peroxidation compared to baseline. Especially in normal-weight women, the extract reduced TBARS levels throughout the entire intervention, whereas in overweight/obese women, TBARS levels were reduced after 4 h compared to baseline levels. Significant differences between the two interventions, placebo and extract, were found only in normal-weight women, suggesting that the extract’s micro-constituents are more effective in those women than in overweight/obese ones. Reduced plasma lipid hydroperoxides were also reported following a meal with 300 mg of grape seed extract compared to the sole meal consumption [27]. MDA, a product of lipid peroxidation, was also reduced compared to placebo due to the 8-week supplementation of oligomeric proanthocyanidins from grape seeds in patients with chronic obstructive pulmonary disease [66].

Following the extract intervention, both BMI groups decreased PC levels at several time points compared to baseline. The extract consumption managed to significantly reduce PC levels by 18–31% after two hours compared to the placebo intervention only in overweight/obese women. No results exist concerning the postprandial effect of grape extract on PC levels. It is likely that the combined elevation of UA levels with the extract’s bioactive compounds may be responsible for the observed reductions in macromolecular damage markers.

During the extract intervention, SOD activity decreased overall. Only in the normal-weight women did the reduction in SOD, by 14–18%, reach significance. No data exist in the literature concerning the postprandial effect of grape extracts on SOD activity in order to compare the results of the present study. It is possible that the observed reductions in SOD activity were induced by the exogenous antioxidants provided by the extract. There are data supporting the idea that flavonoid–metal complexes mimic superoxide dismutase activity, providing an antioxidant defense benefit [67].

Finally, a pilot study was conducted only in four overweight/obese women in order to investigate the long-term effect of the GP extract. The only oxidative stress marker that seemed to be reduced after 4 weeks of consumption was PC. In accordance with these preliminary results, it has been reported that the consumption of 2 g of grape seed powder for 6 months by chronic kidney disease patients was able to reduce PC levels compared to baseline and control [22]. Moreover, the consumption of wine grape pomace flour for 16 weeks reduced PC levels in men with metabolic syndrome [21]. However, a larger study sample is needed in order to evaluate the extract’s long-term actions.

The limitations of the present study include that only a single dose of the GP capsules was administered and that the postprandial effects of the GP extract can only be interpreted in healthy premenopausal women. Moreover, one cannot exclude that a larger study sample would possibly highlight more differences between interventions across the different BMI groups. The strengths of the present study include the measurement of an adequate number of biochemical and oxidative stress biomarkers, as well as the many time points of blood sample collection.

## 5. Conclusions

This study was conducted based on the hypothesis that after the consumption of a high-fat meal, the postprandial elevation of glucose and lipids promotes oxidative stress. Surprisingly, this hypothesis was not proved. It is possible that the rise in the endogenous antioxidant UA prevented the postprandial oxidative response. However, it was highlighted that body weight has an impact on the metabolic and antioxidant responses to the meal as well as to the grape pomace extract’s consumption. In normal-weight women, it seems that grape pomace extract consumption modulates uric acid levels, lipid peroxidation, and superoxide dismutase activity, while in overweight/obese women, its actions are attributed to the modulation of uric acid levels and protein oxidation. These results, although promising, should be replicated in a larger study sample and other populations. Nevertheless, grape pomace extract may be a useful natural antioxidant product that involves the sustainable use of wine by-products.

## Figures and Tables

**Table 1:** Anthropometric features and basic biochemical markers in the two interventions at baseline with respect to BMI categories.

	Placebo BMI < 25 kg/m^2^ (*n* = 11)	Extract BMI < 25 kg/m^2^ (*n* = 11)	Placebo BMI > 25 kg/m^2^ (*n* = 7)	Extract BMI > 25 kg/m^2^ (*n* = 7)
Age (years)	26.5 ± 3.0	26.5 ± 3.0	29.0 ± 2.9	29.0 ± 2.9
Weight (kg)	57.2 (56.2–62.8) ^$^	57 (55.4–61.5) ^$^	81 (66.1–82.4)	81.5 (64.9–83.9)
Height (m)	1.63 ± 0.03	1.63 ± 0.03	1.64 ± 0.07	1.64 ± 0.07
BMI (kg/m^2^)	22.4 ± 1.1 ^$^	22.2 ± 1.1 ^$^	29.5 ± 4.3	29.5 ± 4.6
Waist circumference (cm)	69.6 ± 3.3 ^$^	69.2 ± 4.2 ^$^	82.6 ± 9.7	83.6 ± 8.8
Hip circumference (cm)	96.8 ± 5.2 ^$^	96.6 ± 5.0 ^$^	114.0 ± 9.0	114.4 ± 9.5
Waist-to-hip ratio	0.72 ± 0.04	0.72 ± 0.04	0.72 ± 0.03	0.73 ± 0.03
SBP (mmHg)	108.8 ± 10.0	108.5 ± 11.5	113.6 ± 7.8	117.3 ± 8.2
DBP (mmHg)	63 (61–78)	67 (62–82)	72 (71–80)	72 (70–84)
Heart rate (pulses)	75 (72–92)	80 (66–84)	77 (62–80)	77 (60–95)
Body fat (%)	23.4 (19.8–9.9) ^$^	23.9 (22.7–4.8) ^$^	32.6 (32–42.4)	35.7 (31.7–43.0)
Body fat (kg)	13.3 (11.3–8.8) ^$^	13.9 (12.5–4.5) ^$^	26.5 (21.1–34.9)	27.7 (20.8–36.2)
Body fat-free mass (kg)	44.5 ± 1.7 ^$^	43.7 ± 1.4 ^$^	50.2 ± 4.2	49.9 ± 4.9
Glucose (mg/dL)	82.3 ± 5.4	83.6 ± 6.4	85.1 ± 6.4	87.2 ± 4.5
Insulin (μΙU/mL)	5.5 (4.1–6.3)	6.0 (4.5–6.8)	6.6 (5.5–7.9)	6.6 (5.6–7.7)
HOMA IR	1.1 (0.8–1.3)	1.2 (0.9–1.5)	1.5 (1.2–1.6)	1.5 (1.2–1.6)
Triglycerides (mg/dL)	62.8 ± 17.0	57.9 ± 16.5	63.7 ± 10.1	67.6 ± 8.8
Total cholesterol (mg/dL)	148.4 ± 36.2	153.7 ± 29.0	158.0 ± 34.8	160.2 ± 26.6
HDL-c (mg/dL)	52.2 ± 22.3	50.3 ± 19.2	50.7 ± 10.0	48.0 ± 11.7
LDL-c (mg/dL)	83.7 ± 20.9	91.8 ± 20.3	94.6 ± 34.0	98.7 ± 16.3

Data are presented as means ± SD for normally distributed variables or as medians (25th–75th quartile) for skewed variables. Independent Samples *t*-test or Mann–Whitney U tests were used for the comparisons, respectively. BMI, body mass index; SBP, systolic blood pressure; DBP, diastolic blood pressure; HOMA IR, homeostatic model assessment for insulin resistance; HDL-c, high-density lipoprotein cholesterol; LDL-c, low-density lipoprotein cholesterol. ^$^ *p* < 0.05 compared to BMI > 25 kg/m^2^ participants in the same intervention group.

**Table 2:** Oxidative and endogenous antioxidant biomarkers of the different BMI categories in the two intervention groups at baseline.

	Placebo BMI < 25 kg/m^2^ (*n* = 11)	Extract BMI < 25 kg/m^2^ (*n* = 11)	Placebo BMI > 25 kg/m^2^ (*n* = 7)	Extract BMI > 25 kg/m^2^ (*n* = 7)
Uric acid (mg/dL)	3.6 ± 1.0	4.1 ± 0.8	3.6 ± 1.1	3.7 ± 0.8
TBARS (μM)	2.2 ± 0.8	2.8 ± 2.0	2.4 ± 1.2	2.8 ± 1.9
PC (μmol/μg pr)	0.61 (0.60–0.63)	0.55 (0.50–0.78)	0.56 (0.53–0.62)	0.65 (0.49–0.74)
SOD (U/mg pr)	9.7 ± 5.1	9.7 ± 3.8	11.1 ± 4.5	10.9 ± 4.1
GPx (U/L)	35.6 (31.1–38.0)	32.1 (29.7–36.5)	34.5 (28.9–36.2)	34.1 (29.5–35.7)

Data are presented as means ± SD for normally distributed variables or as medians (25th–75th quartile) for skewed variables. Independent Samples *t*-test or Mann–Whitney U tests were used for the comparisons, respectively. BMI, body mass index; TBARS, thiobarbituric acid reactive substances; PC, protein carbonyls; SOD, superoxide dismutase; GPx, glutathione peroxidase.

**Table 3:** Daily energy, nutrient, and food group intake of the different BMI categories in the two intervention groups at baseline.

	Placebo BMI < 25 kg/m^2^ (*n* = 11)	Extract BMI < 25 kg/m^2^ (*n* = 11)	Placebo BMI > 25 kg/m^2^ (*n* = 7)	Extract BMI > 25 kg/m^2^ (*n* = 7)
Energy intake (kcal)	1539.4 (1389.1–2565.1)	1546.0 ^$^ (1344.2–1893.7)	1973.1 (1571.2–2362.5)	1997.8 (1813.2–2520.3)
Carbohydrates (% of total energy)	39.3 ± 9.6	34.2 ± 5.3	34.9 ± 7.8	38.3 ± 7.6
Proteins (% of total energy)	17.0 ± 4.1	19.7 ± 5.3	18.0 ± 5.1	15.9 ± 1.4
Lipids (% of total energy)	39.4 ± 7.5	44.3 ± 5.7	45.4 ± 5.6	44.2 ± 6.2
Protein intake/body weight (g/kg)	1.3 ± 0.4	1.4 ± 0.4 ^$^	1.1 ± 0.4	1.0 ± 0.2
SFA (% of total energy)	12.9 ± 3.0	13.2 ± 2.6	14.6 ± 2.1	15.3 ± 2.2
MUFA (% of total energy)	16.0 ± 5.1 ^$^	19.1 ± 5.9	20.7 ± 3.4	18.6 ± 3.8
PUFA (% of total energy)	5.7 ± 2.6	7.1 ± 2.3	7.3 ± 2.1	6.7 ± 1.6
Fiber (g)	17.7 (12.0–21.3)	11.7 (11.1–17.1)	13.0 (12.0–13.9)	13.1 (11.5–26.0)
Vitamin A (IU)	1479.9 (1109.9–5090.7)	4068 (1782.9–14012.2)	5352.1 (3673.4–8264.2)	3127.2 (2634.0–6482.8)
Vitamin C (mg)	28.5 (13.3–37.8) ^$^	49.7 (19.8–78.0) ^$^	76.5 (32.3–96.1)	108.9 (82.7–229.2)
Dairy (servings/day)	1.0 (0.4–2.6)	0.7 (0.6–2.6)	0.2 (0.0–2.1)	0.2 (0.0–1.3)
Fruits and vegetables (servings/d)	1.6 ± 1.2	2.3 ± 1.4	2.7 ± 1.8	3.6 ± 3.1
Legumes (servings/day)	0.0 (0.0–0.4)	0.0 (0.0–0.0)	0.0 (0.0–0.0)	0.0 (0.0–0.3)
Fish (servings/day)	0.0 (0.0–0.7)	0.6 (0.0–0.7)	0.1 (0.0–0.3)	0.0 (0.0–0.3)
Red and processed meat (servings/day)	1.1 ± 0.9 ^$^	1.4 ± 1.2	2.5 ± 1.2	2.1 ± 0.7
Chicken/turkey (servings/day)	1.2 ± 1.0	0.8 ± 0.5	0.7 ± 0.6	0.5 ± 0.8
Whole grains (servings/day)	0.8 (0.4–1.3)	0.2 (0.0–1.7)	0.5 (0.3–2.5)	1.4 (0.4–2.2)
Refined grains (servings/day)	3.5 ± 2.1	2.5 ± 1.5	3.6 ± 1.7	3.4 ± 1.5
Nuts (servings/day)	0.3 ± 0.4	0.3 ± 0.4	0.2 ± 0.2	0.4 ± 0.4
Sweets (servings/day)	0.5 (0.2–0.9)	0.6 (0.0–1.1)	0.4 (0.0–1.8)	0.9 (0.8–1.5)
Physical activity levels	1.4 ± 0.2 ^$^	1.4 ± 0.2 ^$^	1.7 ± 0.2	1.8 ± 0.2

Data are presented as means ± SD for normally distributed variables or as medians (25th–75th quartile) for skewed variables. Independent Samples *t*-test or Mann–Whitney U tests were used for the comparisons, respectively. BMI, body mass index; SFA, saturated fatty acids; MUFA, monounsaturated fatty acids; PUFA, polyunsaturated fatty acids. ^$^ *p* < 0.05 compared to BMI > 25 kg/m^2^ participants in the same intervention group.

**Table 4:** Percentage of change in basic biochemical markers in the two intervention groups.

	−15	0	30	60	90	120	150	180	210	240	300	360	^^^ p_time_ ^^^ p_time*trial_ ^^^ p_trial_
%Glucose-Placebo	100	104.3 ± 11.4	112.5 ± 23.4 ^#^	92.6 ± 14.5 ^#^	98.4 ± 14.6	106.0 ± 17.3	103.5 ± 11.1	101.7 ± 13.3	101.1 ± 9.7	100.5 ± 9.8	100.7 ± 9.2	99.0 ± 9.4	0.06
%Glucose-Extract	100	104.3 ± 9.7	112.8 ± 15.0 ^#^	97.8 ± 10.7	103.6 ± 15.6	104.7 ± 14.7	102.5 ± 7.3	102.2 ± 9.8	102.5 ± 7.7	100.8 ± 10.8	98.1 ± 8.8	94.7 ± 8.9 ^#^	0.69
^†^ p_P-E_	-	0.98	0.95	0.13	0.27	0.65	0.69	0.87	0.52	0.92	0.27	0.08	0.86
%Insulin-Placebo	100	-	706.1 ± 472.1 ^#^	449.7 ± 171.6 ^#^	420.1 ± 196.1 ^#^	474.2 ± 210.2 ^#^	394.1 ± 173.9 ^#^	-	-	-	-	-	<0.01
%Insulin-Extract	100	-	689.9 ± 281.7 ^#^	584.9 ± 328.2 ^#^	457.6 ± 194.5 ^#^	459.3 ± 186.9 ^#^	369.2 ± 110.1 ^#^	-	-	-	-	-	0.44
^†^ p_P-E_	-	-	0.85	0.04	0.40	0.69	0.58	-	-	-	-	-	0.67
%TG-Placebo	100	97.1 ± 10.0	125.2 ± 23.7 ^#^	154.2 ± 37.5 ^#^	171.7 ± 48.5 ^#^	195.3 ± 63.4 ^#^	195.5 ± 79.9 ^#^	188.7 ± 65.1 ^#^	178.8 ± 65.8 ^#^	159.4 ± 52.1 ^#^	168.1 ± 71.7 ^#^	163.8 ± 58.2 ^#^	<0.01
%TG-Extract	100	97.5 ± 10.5	120.0 ± 20.9 ^#^	152.5 ± 31.4 ^#^	173.1 ± 32.7 ^#^	180.0 ± 37.4 ^#^	176.3 ± 38.5 ^#^	175.2 ± 59.6 ^#^	165.4 ± 43.4 ^#^	167.1 ± 41.8 ^#^	136.7 ± 22.5 ^#^	140.6 ± 34.3 ^#^	0.29
^†^ p_P-E_	-	0.87	0.34	0.82	0.90	0.35	0.31	0.50	0.44	0.60	0.04	0.11	0.41
%Total cholesterol-Placebo	100	98.9 ± 7.0	101.9 ± 7.5	100.5 ± 8.9	106.2 ± 7.3	102.3 ± 9.1	105.5 ± 10.2	100.0 ± 12.1	102.1 ± 8.2	98.0 ± 6.8	100.7 ± 11.1	102.7 ± 8.3	0.13
%Total cholesterol-Extract	100	99.4 ± 4.8	100.9 ± 4.9	99.6 ± 5.6	103.4 ± 7.4	98.5 ± 6.6	102.2 ± 8.1	97.4 ± 7.4	96.6 ± 6.7	97.4 ± 6.8	96.8 ± 5.4	101.3 ± 5.5	0.41
^†^ p_P-E_	-	0.82	0.69	0.68	0.21	0.15	0.20	0.31	0.01	0.74	0.12	0.54	0.25
%HDL-c-Placebo	100	103.2 ± 8.0	99.6 ± 7.3	102.0 ± 9.7	97.4 ± 10.4	97.1 ± 10.9	95.1 ± 12.0	96.3 ± 11.3	95.5 ± 10.2	97.6 ± 9.0	99.7 ± 10.7	99.8 ± 10.3	<0.01
%HDL-c-Extract	100	100.2 ± 4.8	101.2 ± 8.2	98.6 ± 7.7	95.9 ± 7.5	94.7 ± 8.6 ^#^	94.7 ± 7.9 ^#^	92.0 ± 10.1 ^#^	92.1 ± 8.2 ^#^	94.4 ± 10.1 ^#^	96.1 ± 7.5 ^#^	101.1 ± 10.0	0.27
^†^ p_P-E_	-	0.17	0.53	0.27	0.59	0.45	0.90	0.26	0.31	0.36	0.27	0.74	0.45
%LDL-c-Placebo	100	97.5 ± 11.0	99.6 ± 10.2	91.7 ± 13.0 ^#^	102.0 ± 11.8	91.4 ± 13.1 ^#^	98.1 ± 9.4	90.2 ± 17.3 ^#^	94.9 ± 12.3	90.1 ± 13.5 ^#^	91.9 ± 17.7	96.8 ± 14.6	0.03
%LDL-c-Extract	100	99.3 ± 7.0	97.0 ± 7.8	93.0 ± 7.8 ^#^	97.7 ± 9.9	89.0 ± 9.8 ^#^	95.6 ± 8.9 ^#^	88.7 ± 8.8 ^#^	88.8 ± 6.5 ^#^	88.4 ± 8.7 ^#^	91.8 ± 8.0 ^#^	96.4 ± 7.9	0.68
^†^ p_P-E_	-	0.62	0.46	0.73	0.30	0.47	0.42	0.70	0.08	0.64	0.99	0.92	0.51

For normally distributed variables, data are presented as means ± SD. Repeated measures ANOVA was used for the comparisons (p_time_, p_trial_, p_time*trial_). Paired samples *t*-test was used to compare placebo vs. extract at each time point (p_P-E_) and each time point to the baseline within each intervention. TG, triglycerides; HDL-c, high-density lipoprotein cholesterol; LDL-c, low-density lipoprotein cholesterol. ^^^ *p* trend from RMANOVA, ^†^ *p* value from paired samples *t*-test, ^#^ *p* ≤ 0.05 compared to baseline.

**Table 5:** Percentage of change in oxidative and endogenous antioxidant biomarkers in the two intervention groups.

	−15	0	30	60	90	120	150	180	210	240	300	360	^^^ p_time_ ^^^ p_time*trial_ ^^^ p_trial_	^§†^ p_time_
%UA-Placebo	100	102.4 ± 6.1	105.0 ± 7.5 ^#^	112.9 ± 12.9 ^#^	118.0 ± 12.8 ^#^	122.7 ± 25.2 ^#^	114.5 ± 23.0 ^#^	108.9 ± 25.3	102.5 ± 18.7	98.2 ± 13.5	98.1 ± 23.6	98.4 ± 15.5	<0.01	-
%UA-Extract	100	99.7 ± 7.7	102.6 ± 6.9	111.0 ± 14.1 ^#^	117.0 ± 10.3 ^#^	111.6 ± 16.3 ^#^	113.5 ± 18.8 ^#^	109.0 ± 19.8	104.2 ± 16.9	104.3 ± 15.3	92.6 ± 9.3 ^#^	93.5 ± 10.4 ^#^	0.48	-
^†^p_P-E_	-	0.33	0.36	0.63	0.76	0.29	0.58	0.99	0.71	0.17	0.21	0.14	0.62	-
%TBARS-Placebo	100	-	-	97.8 ± 14.2	-	95.8 ± 13.7	-	91.7 ± 18.0	-	91.5 ± 25.7	82.1 ± 19.8 ^#^	78.0 ± 13.3 ^#^	<0.01	-
%TBARS-Extract	100	-	-	97.9 ± 17.6	-	94.0 ± 10.7 ^#^	-	84.2 ± 11.6 ^#^	-	79.4 ± 16.2 ^#^	74.3 ± 14.9 ^#^	77.5 ± 17.4 ^#^	0.14	-
^†^ p_P-E_	-	-	-	0.97	-	0.60	-	0.08	-	0.08	0.08	0.91	0.27	-
%PC-Placebo	100	98.0 ± 13.7	109.0 ± 20.8	103.7 ± 15.4	101.9 ± 15.6	104.1 ± 20.6	97.0 ± 12.6	99.0 ± 14.1	101.1 ± 20.9	104.0 ± 15.3	101.9 ± 20.6	110.4 ± 23.4	0.74	-
%PC-Extract	100	90.6 ± 6.8 ^#^	92.8 ± 13.5 ^#^	93.9 ± 13.3	89.1 ± 10.2 ^#^	91.4 ± 12.6 ^#^	95.1 ± 17.7	91.5 ± 12.2 ^#^	98.2 ± 20.6	92.6 ± 16.5	89.3 ± 11.5 ^#^	87.6 ± 15.5 ^#^	0.03	-
^†^ p_P-E_	-	0.06	0.02	0.08	0.02	0.06	0.68	0.08	0.69	0.06	0.04	<0.01	<0.01	-
%SOD-Placebo	100	-	-	102.7 (81.1–119.2)	-	99.9 (77.2–151.1)	-	88.6 (68.2–122.2)	90.4 (82.2–103.9)	92.6 (79.6–109.3)	92.0 (71.2–108.0)	89.4 (63.4–103.9)	-	0.25
%SOD-Extract	100	-	-	89.1 (67.9–109.1)	-	77.5 (70.9–97.4) ^#^	-	89.5 (65.5–102.6) ^#^	87.7 (70.5–102.4)	82.6 (70.4–128.1)	82.0 (71.6–104.5)	101.6 (68.4–117.2)	-	0.04
^†^ p_P-E_	-	-	-	0.03	-	0.01	-	0.81	0.55	0.50	0.44	0.21		
%GPx-Placebo	100	103.7 ± 4.0 ^#^	99.1 ± 4.8	99.6 ± 5.2	100.2 ± 7.8	97.8 ± 6.7	101.0 ± 9.1	99.1 ± 5.7	100.2 ± 9.9	99.3 ± 8.4	101.1 ± 7.0	102.5 ± 5.8	0.49	-
%GPx-Extract	100	104.6 ± 5.5 ^#^	101.7 ± 4.8	99.9 ± 6.2	99.7 ± 8.3	99.1 ± 7.4	102.6 ± 8.6	101.0 ± 8.9	98.8 ± 8.4	100.9 ± 7.4	101.7 ± 6.6	103.7 ± 9.3	0.91	-
^†^ p_P-E_	-	0.62	0.12	0.82	0.85	0.62	0.60	0.41	0.67	0.47	0.79	0.68	0.58	-

For normally distributed variables, data are presented as means ± SD. Repeated measures ANOVA was used for the comparisons (p_time_, p_trial_, p_time*trial_). Paired samples *t*-test was used to compare each time point to the other intervention (p_P-E_) and to the baseline. For skewed variables, data are presented as the median (25th–75th quartile). Friedman’s 2-way ANOVA by ranks was used for the estimation of the time effect in each intervention group (^§^ p_time_) and the Wilcoxon paired test was used for differences compared to the baseline. Trial-related pairwise comparisons were performed using the Wilcoxon paired test (p_P-E_). UA, uric acid; TBARS, thiobarbituric acid reactive substances; PC, protein carbonyls; SOD, superoxide dismutase; GPx, glutathione peroxidase. ^^^ *p* trend from RMANOVA; ^†^ *p*-value from paired samples *t*-test, Wilcoxon paired test, Friedman; ^#^ *p* ≤ 0.05 compared to baseline.

**Table 6:** Percentage of change in basic biochemical and oxidative stress markers in the two long-term interventions.

	Placebo (*n* = 4)	Extract (*n* = 4)
%Glucose	100.2 (96.7–106.2)	102.4 (97.0–104.6)
%Insulin	94.6 (81.8–105.4)	105.8 (61.7–121.0)
%Triglycerides	122.0 (82.3–133.3)	85.8 (76.8–109.8)
%Total Cholesterol	93.6 (89.2–111.4)	89.8 (85.0–94.1)
%HDL-c	100.7 (96.3–124.0)	99.1 (82.8–126.0)
%LDL-c	81.0 (70.6–114.4)	81.7 (65.4–102.7)
%Uric acid	104.8 (74.5–141.2)	101.8 (87.4–131.2)
%TBARS	116.1 (91.2–165.2)	102.4 (79.8–137.6)
%Protein Carbonyls	97.3 (74.9–144.3)	87.6 (78.5–98.0)
%SOD	86.1 (70.5–127.1)	100.1 (79.1–146.9)
%GPx	102.4 (95.3–106.5)	100.1 (95.3–103.4)

Data are presented as medians (25th–75th quartile). Wilcoxon paired test was used to compare values with the baseline and between interventions. HDL-c, high-density lipoprotein cholesterol; LDL-c, low-density lipoprotein cholesterol; TBARS, thiobarbituric acid reactive substances; SOD, superoxide dismutase; GPx, glutathione peroxidase.

## Data Availability

Not applicable.

## Supplementary Materials

The following supporting information can be downloaded at: https://www.mdpi.com/article/10.3390/nu15010156/s1, Table S1: Percentage of change in basic biochemical markers in the two intervention groups for participants with BMI < 25 kg/m^2^; Table S2: Percentage of change in basic biochemical markers in the two intervention groups for participants with BMI > 25 kg/m^2^; Table S3: Percentage of change in oxidative and endogenous antioxidant biomarkers in the two intervention groups for participants with BMI < 25 kg/m^2^; Table S4: Percentage of change in oxidative and endogenous antioxidant biomarkers in the two intervention groups for participants with BMI > 25 kg/m^2^.

## References

[1] Zhao Y., Liu L., Yang S., Liu G., Pan L., Gu C., Wang Y., Li D., Zhao R., Wu M. (2021). Mechanisms of Atherosclerosis Induced by Postprandial Lipemia. Front. Cardiovasc. Med..

[2] Wallace J.P., Johnson B., Padilla J., Mather K. (2010). Postprandial Lipaemia, Oxidative Stress and Endothelial Function: A Review. Int. J. Clin. Pract..

[3] Meessen E.C.E., Warmbrunn M.V., Nieuwdorp M., Soeters M.R. (2019). Human Postprandial Nutrient Metabolism and Low-Grade Inflammation: A Narrative Review. Nutrients.

[4] Bloomer R.J., Fisher-Wellman K.H. (2009). Systemic Oxidative Stress Is Increased to a Greater Degree in Young, Obese Women Following Consumption of a High Fat Meal. Oxid. Med. Cell. Longev..

[5] Murray M., Selby-Pham S., Colton B.-L., Bennett L., Williamson G., Dordevic A.L. (2021). Does Timing of Phytonutrient Intake Influence the Suppression of Postprandial Oxidative Stress? A Systematic Literature Review. Redox Biol..

[6] Islam M.A., Alam F., Solayman M., Khalil M.I., Kamal M.A., Gan S.H. (2016). Dietary Phytochemicals: Natural Swords Combating Inflammation and Oxidation-Mediated Degenerative Diseases. Oxid. Med. Cell. Longev..

[7] St Leger A.S., Cochrane A.L., Moore F. (1979). Factors Associated with Cardiac Mortality in Developed Countries with Particular Reference to the Consumption of Wine. Lancet.

[8] Fragopoulou E., Choleva M., Antonopoulou S., Demopoulos C.A. (2018). Wine and Its Metabolic Effects. A Comprehensive Review of Clinical Trials. Metabolism..

[9] Covas M.I., Gambert P., Fitó M., de la Torre R. (2010). Wine and Oxidative Stress: Up-to-Date Evidence of the Effects of Moderate Wine Consumption on Oxidative Damage in Humans. Atherosclerosis.

[10] Castaldo L., Narváez A., Izzo L., Graziani G., Gaspari A., Minno G.D., Ritieni A. (2019). Red Wine Consumption and Cardiovascular Health. Molecules.

[11] Fragopoulou E., Demopoulos C.A., Antonopoulou S. (2009). Lipid Minor Constituents in Wines. A Biochemical Approach in the French Paradox. Int. J. Wine Res..

[12] Xanthopoulou M.N., Kalathara K., Melachroinou S., Arampatzi-Menenakou K., Antonopoulou S., Yannakoulia M., Fragopoulou E. (2017). Wine Consumption Reduced Postprandial Platelet Sensitivity against Platelet Activating Factor in Healthy Men. Eur. J. Nutr..

[13] Argyrou C., Vlachogianni I., Stamatakis G., Demopoulos C.A., Antonopoulou S., Fragopoulou E. (2017). Postprandial Effects of Wine Consumption on Platelet Activating Factor Metabolic Enzymes. Prostaglandins Other Lipid Mediat..

[14] Fragopoulou E., Argyrou C., Detopoulou M., Tsitsou S., Seremeti S., Yannakoulia M., Antonopoulou S., Kolovou G., Kalogeropoulos P. (2021). The Effect of Moderate Wine Consumption on Cytokine Secretion by Peripheral Blood Mononuclear Cells: A Randomized Clinical Study in Coronary Heart Disease Patients. Cytokine.

[15] Choleva M., Argyrou C., Detopoulou M., Donta M.-E., Gerogianni A., Moustou E., Papaemmanouil A., Skitsa C., Kolovou G., Kalogeropoulos P. (2022). Effect of Moderate Wine Consumption on Oxidative Stress Markers in Coronary Heart Disease Patients. Nutrients.

[16] Gerardi G., Cavia-Saiz M., Muñiz P. (2022). From Winery By-Product to Healthy Product: Bioavailability, Redox Signaling and Oxidative Stress Modulation by Wine Pomace Product. Crit. Rev. Food Sci. Nutr..

[17] Fontana A.R., Antoniolli A., Bottini R. (2013). Grape Pomace as a Sustainable Source of Bioactive Compounds: Extraction, Characterization, and Biotechnological Applications of Phenolics. J. Agric. Food Chem..

[18] Argani H., Ghorbanihaghjo A., Vatankhahan H., Rashtchizadeh N., Raeisi S., Ilghami H. (2016). The Effect of Red Grape Seed Extract on Serum Paraoxonase Activity in Patients with Mild to Moderate Hyperlipidemia. Sao Paulo Med. J. Rev. Paul. Med..

[19] Han H.J., Jung U.J., Kim H.-J., Cho S.-J., Kim A.H., Han Y., Choi M.-S. (2016). Combined Supplementation with Grape Pomace and Omija Fruit Ethanol Extracts Dose-Dependently Improves Body Composition, Plasma Lipid Profiles, Inflammatory Status, and Antioxidant Capacity in Overweight and Obese Subjects. J. Med. Food.

[20] Yubero N., Sanz-Buenhombre M., Guadarrama A., Villanueva S., Carrión J.M., Larrarte E., Moro C. (2013). LDL Cholesterol-Lowering Effects of Grape Extract Used as a Dietary Supplement on Healthy Volunteers. Int. J. Food Sci. Nutr..

[21] Urquiaga I., D’Acuña S., Pérez D., Dicenta S., Echeverría G., Rigotti A., Leighton F. (2015). Wine Grape Pomace Flour Improves Blood Pressure, Fasting Glucose and Protein Damage in Humans: A Randomized Controlled Trial. Biol. Res..

[22] Turki K., Charradi K., Boukhalfa H., Belhaj M., Limam F., Aouani E. (2016). Grape Seed Powder Improves Renal Failure of Chronic Kidney Disease Patients. EXCLI J..

[23] De Groote D., Van Belleghem K., Devière J., Van Brussel W., Mukaneza A., Amininejad L. (2012). Effect of the Intake of Resveratrol, Resveratrol Phosphate, and Catechin-Rich Grape Seed Extract on Markers of Oxidative Stress and Gene Expression in Adult Obese Subjects. Ann. Nutr. Metab..

[24] van Mierlo L.A.J., Zock P.L., van der Knaap H.C.M., Draijer R. (2010). Grape Polyphenols Do Not Affect Vascular Function in Healthy Men. J. Nutr..

[25] Sapwarobol S., Adisakwattana S., Changpeng S., Ratanawachirin W., Tanruttanawong K., Boonyarit W. (2012). Postprandial Blood Glucose Response to Grape Seed Extract in Healthy Participants: A Pilot Study. Pharmacogn. Mag..

[26] Costabile G., Vitale M., Luongo D., Naviglio D., Vetrani C., Ciciola P., Tura A., Castello F., Mena P., Del Rio D. (2019). Grape Pomace Polyphenols Improve Insulin Response to a Standard Meal in Healthy Individuals: A Pilot Study. Clin. Nutr..

[27] Natella F., Belelli F., Gentili V., Ursini F., Scaccini C. (2002). Grape Seed Proanthocyanidins Prevent Plasma Postprandial Oxidative Stress in Humans. J. Agric. Food Chem..

[28] Bloomer R.J., Ferebee D.E., Fisher-Wellman K.H., Quindry J.C., Schilling B.K. (2009). Postprandial Oxidative Stress: Influence of Sex and Exercise Training Status. Med. Sci. Sports Exerc..

[29] Choleva M., Boulougouri V., Panara A., Panagopoulou E., Chiou A., Thomaidis N.S., Antonopoulou S., Fragopoulou E. (2019). Evaluation of Anti-Platelet Activity of Grape Pomace Extracts. Food Funct..

[30] Bountziouka V., Bathrellou E., Giotopoulou A., Katsagoni C., Bonou M., Vallianou N., Barbetseas J., Avgerinos P.C., Panagiotakos D.B. (2012). Development, Repeatability and Validity Regarding Energy and Macronutrient Intake of a Semi-Quantitative Food Frequency Questionnaire: Methodological Considerations. Nutr. Metab. Cardiovasc. Dis..

[31] Panagiotakos D.B., Pitsavos C., Arvaniti F., Stefanadis C. (2007). Adherence to the Mediterranean Food Pattern Predicts the Prevalence of Hypertension, Hypercholesterolemia, Diabetes and Obesity, among Healthy Adults; the Accuracy of the MedDietScore. Prev. Med..

[32] Kavouras S.A., Maraki M.I., Kollia M., Gioxari A., Jansen L.T., Sidossis L.S. (2016). Development, Reliability and Validity of a Physical Activity Questionnaire for Estimating Energy Expenditure in Greek Adults. Sci. Sports.

[33] Detopoulou P., Nomikos T., Fragopoulou E., Antonopoulou S., Kotroyiannis I., Vassiliadou C., Panagiotakos D.B., Chrysohoou C., Pitsavos C., Stefanadis C. (2009). Platelet Activating Factor (PAF) and Activity of Its Biosynthetic and Catabolic Enzymes in Blood and Leukocytes of Male Patients with Newly Diagnosed Heart Failure. Clin. Biochem..

[34] Bradford M.M. (1976). A Rapid and Sensitive Method for the Quantitation of Microgram Quantities of Protein Utilizing the Principle of Protein-Dye Binding. Anal. Biochem..

[35] Jentzsch A.M., Bachmann H., Fürst P., Biesalski H.K. (1996). Improved Analysis of Malondialdehyde in Human Body Fluids. Free Radic. Biol. Med..

[36] McCord J.M. (2001). Analysis of Superoxide Dismutase Activity. Curr. Protoc. Toxicol..

[37] Paglia D.E., Valentine W.N. (1967). Studies on the Quantitative and Qualitative Characterization of Erythrocyte Glutathione Peroxidase. J. Lab. Clin. Med..

[38] Dimina L., Mariotti F. (2019). The Postprandial Appearance of Features of Cardiometabolic Risk: Acute Induction and Prevention by Nutrients and Other Dietary Substances. Nutrients.

[39] Fappi A., Mittendorfer B. (2020). Different Physiological Mechanisms Underlie an Adverse Cardiovascular Disease Risk Profile in Men and Women. Proc. Nutr. Soc..

[40] Cremonini E., Daveri E., Iglesias D.E., Kang J., Wang Z., Gray R., Mastaloudis A., Kay C.D., Hester S.N., Wood S.M. (2022). A Randomized Placebo-Controlled Cross-over Study on the Effects of Anthocyanins on Inflammatory and Metabolic Responses to a High-Fat Meal in Healthy Subjects. Redox Biol..

[41] Bantle A.E., Thomas W., Bantle J.P. (2008). Metabolic Effects of Alcohol in the Form of Wine in Persons with Type 2 Diabetes Mellitus. Metabolism.

[42] Lacroix S., Rosiers C.D., Tardif J.-C., Nigam A. (2012). The Role of Oxidative Stress in Postprandial Endothelial Dysfunction. Nutr. Res. Rev..

[43] Title L.M., Cummings P.M., Giddens K., Nassar B.A. (2000). Oral Glucose Loading Acutely Attenuates Endothelium-Dependent Vasodilation in Healthy Adults without Diabetes: An Effect Prevented by Vitamins C and E. J. Am. Coll. Cardiol..

[44] Williams M.J.A., Sutherland W.H.F., McCormick M.P., de Jong S.A., Walker R.J., Wilkins G.T. (1999). Impaired Endothelial Function Following a Meal Rich in Used Cooking Fat. J. Am. Coll. Cardiol..

[45] Fisher-Wellman K., Bloomer R. (2009). Macronutrient Specific Postprandial Oxidative Stress: Relevance to the Development of Insulin Resistance. Curr. Diabetes Rev..

[46] Bloomer R.J., Kabir M.M., Marshall K.E., Canale R.E., Farney T.M. (2010). Postprandial Oxidative Stress in Response to Dextrose and Lipid Meals of Differing Size. Lipids Health Dis..

[47] Kawano H., Motoyama T., Hirashima O., Hirai N., Miyao Y., Sakamoto T., Kugiyama K., Ogawa H., Yasue H. (1999). Hyperglycemia Rapidly Suppresses Flow-Mediated Endothelium-Dependent Vasodilation of Brachial Artery. J. Am. Coll. Cardiol..

[48] Serin O., Konukoglu D., Firtina S., Mavis O. (2007). Serum Oxidized Low Density Lipoprotein, Paraoxonase 1 and Lipid Peroxidation Levels during Oral Glucose Tolerance Test. Horm. Metab. Res. Horm. Stoffwechselforschung Horm. Metab..

[49] Montes-Nieto R., Insenser M., Murri M., Fernández-Durán E., Ojeda-Ojeda M., Martínez-García M.Á., Luque-Ramírez M., Escobar-Morreale H.F. (2017). Plasma Thiobarbituric Acid Reactive Substances (TBARS) in Young Adults: Obesity Increases Fasting Levels Only in Men Whereas Glucose Ingestion, and Not Protein or Lipid Intake, Increases Postprandial Concentrations Regardless of Sex and Obesity. Mol. Nutr. Food Res..

[50] Bae J.-H., Schwemmer M., Lee I.-K., Lee H.-J., Park K.-R., Kim K.-Y., Bassenge E. (2003). Postprandial Hypertriglyceridemia-Induced Endothelial Dysfunction in Healthy Subjects Is Independent of Lipid Oxidation. Int. J. Cardiol..

[51] Tushuizen M.E. (2005). Postprandial Dysmetabolism and Cardiovascular Disease in Type 2 Diabetes. Postgrad. Med. J..

[52] Anderson R.A., Evans L.M., Ellis G.R., Khan N., Morris K., Jackson S.K., Rees A., Lewis M.J., Frenneaux M.P. (2006). Prolonged Deterioration of Endothelial Dysfunction in Response to Postprandial Lipaemia Is Attenuated by Vitamin C in Type 2 Diabetes. Diabet. Med..

[53] Anderson R.A., Evans M.L., Ellis G.R., Graham J., Morris K., Jackson S.K., Lewis M.J., Rees A., Frenneaux M.P. (2001). The Relationships between Post-Prandial Lipaemia, Endothelial Function and Oxidative Stress in Healthy Individuals and Patients with Type 2 Diabetes. Atherosclerosis.

[54] Neri S., Calvagno S., Mauceri B., Misseri M., Tsami A., Vecchio C., Mastrosimone G., Di Pino A., Maiorca D., Judica A. (2010). Effects of Antioxidants on Postprandial Oxidative Stress and Endothelial Dysfunction in Subjects with Impaired Glucose Tolerance and Type 2 Diabetes. Eur. J. Nutr..

[55] Neri S., Signorelli S.S., Torrisi B., Pulvirenti D., Mauceri B., Abate G., Ignaccolo L., Bordonaro F., Cilio D., Calvagno S. (2005). Effects of Antioxidant Supplementation on Postprandial Oxidative Stress and Endothelial Dysfunction: A Single-Blind, 15-Day Clinical Trial in Patients with Untreated Type 2 Diabetes, Subjects with Impaired Glucose Tolerance, and Healthy Controls. Clin. Ther..

[56] Johnson B.D., Padilla J., Harris R.A., Wallace J.P. (2011). Vascular Consequences of a High-Fat Meal in Physically Active and Inactive Adults. Appl. Physiol. Nutr. Metab..

[57] Ide T., Tsutsui H., Ohashi N., Hayashidani S., Suematsu N., Tsuchihashi M., Tamai H., Takeshita A. (2002). Greater Oxidative Stress in Healthy Young Men Compared With Premenopausal Women. Arterioscler. Thromb. Vasc. Biol..

[58] Spallarossa P., Garibaldi S., Barisione C., Ghigliotti G., Altieri P., Tracchi I., Fabbi P., Barsotti A., Brunelli C. (2008). Postprandial Serum Induces Apoptosis in Endothelial Cells: Role of Polymorphonuclear-Derived Myeloperoxidase and Metalloproteinase-9 Activity. Atherosclerosis.

[59] Cardona F., Túnez I., Tasset I., Garrido-Sánchez L., Collantes E., Tinahones F.J. (2008). Circulating Antioxidant Defences Are Decreased in Healthy People after a High-Fat Meal. Br. J. Nutr..

[60] Urquiaga I., Ávila F., Echeverria G., Perez D., Trejo S., Leighton F. (2017). A Chilean Berry Concentrate Protects against Postprandial Oxidative Stress and Increases Plasma Antioxidant Activity in Healthy Humans. Oxid. Med. Cell. Longev..

[61] Mirończuk-Chodakowska I., Witkowska A.M., Zujko M.E. (2018). Endogenous Non-Enzymatic Antioxidants in the Human Body. Adv. Med. Sci..

[62] Wu B., Roseland J.M., Haytowitz D.B., Pehrsson P.R., Ershow A.G. (2019). Availability and Quality of Published Data on the Purine Content of Foods, Alcoholic Beverages, and Dietary Supplements. J. Food Compos. Anal..

[63] Miglio C., Peluso I., Raguzzini A., Villaño D.V., Cesqui E., Catasta G., Toti E., Serafini M. (2014). Fruit Juice Drinks Prevent Endogenous Antioxidant Response to High-Fat Meal Ingestion. Br. J. Nutr..

[64] Edirisinghe I., Randolph J., Cheema M., Tadapaneni R., Park E., Burton-Freeman B., Kappagoda T. (2013). Effect of Grape Seed Extract on Postprandial Oxidative Status and Metabolic Responses in Men and Women with the Metabolic Syndrome—Randomized, Cross-over, Placebo-Controlled Study. Funct. Foods Health Dis..

[65] Wang Y., Zhu J.X., Kong L.D., Yang C., Cheng C.H.K., Zhang X. (2004). Administration of Procyanidins from Grape Seeds Reduces Serum Uric Acid Levels and Decreases Hepatic Xanthine Dehydrogenase/Oxidase Activities in Oxonate-Treated Mice. Basic Clin. Pharmacol. Toxicol..

[66] Lu M.-C., Yang M.-D., Li P.-C., Fang H.-Y., Huang H.-Y., Chan Y.-C., Bau D.-T. (2018). Effect of Oligomeric Proanthocyanidin on the Antioxidant Status and Lung Function of Patients with Chronic Obstructive Pulmonary Disease. In Vivo.

[67] Kostyuk V.A., Potapovich A.I., Strigunova E.N., Kostyuk T.V., Afanas’ev I.B. (2004). Experimental Evidence that Flavonoid Metal Complexes May Act as Mimics of Superoxide Dismutase. Arch. Biochem. Biophys..

