# Supplementary material for: Postprandial Metabolic and Oxidative Stress Responses to Grape Pomace Extract in Healthy Normal and Overweight/Obese Women: A Randomized, Double-Blind, Placebo-Controlled Crossover Study
Source: Nutrients. 2022 Dec 29;15(1):156. doi: 10.3390/nu15010156 (PMC9824782; doi:10.3390/nu15010156)
Supplement: Supplementary file 1 [file nutrients-15-00156-s001.zip › Supplementary Tables.pdf]

Supplementary Table S1: Percentage of change in basic biochemical markers in the two intervention groups for participants with BMI < 25kg/m^2.

	-15	0	30	60	90	120	150	180	210	240	300	360	$^{\wedge}p_{time}$ / $^{\wedge}p_{time*trial}$ / $^{\wedge}p_{trial}$
%Glucose -Placebo	100	102.9± 7.8	106.2± 19.3	91.8± 14.8	95.6± 16.2	104.4± 20.8	102.7± 12.9	101.0± 13.5	101.9± 11.7	100.0± 11.9	102.5± 10.1	101.5± 9.7	0.04
%Glucose -Extract	100	100.6± 7.3	110.9± 14.1$^{\#}$	96.6± 11.3	104.0± 14.8	105.9± 17.2	103.0± 6.7	103.0± 11.6	103.3± 8.3	104.7± 11.5	101.8± 8.4	98.0± 7.8	0.70
$^{+}p_{P-E}$	-	0.38	0.31	0.33	0.22	0.70	0.91	0.63	0.61	0.32	0.80	0.32	0.48
%Insulin-Placebo	100	-	710.9± 555.4$^{\#}$	468.4± 193.0$^{\#}$	453.0± 210.5$^{\#}$	496.5± 245.4$^{\#}$	409.9± 219.5$^{\#}$	-	-	-	-	-	<0.01
%Insulin-Extract	100	-	715.2± 322.1$^{\#}$	564.9± 246.8$^{\#}$	446.9± 146.2$^{\#}$	474.7± 175.7$^{\#}$	376.9± 105.9$^{\#}$	-	-	-	-	-	0.77
$^{+}p_{P-E}$	-	-	0.97	0.05	0.92	0.65	0.63	-	-	-	-	-	0.91
%TG-Placebo	100	99.4± 10.3	128.0± 27.5$^{\#}$	161.8± 44.5$^{\#}$	187.1± 54.8$^{\#}$	212.7± 75.1$^{\#}$	219.0± 95.3$^{\#}$	208.0± 76.1$^{\#}$	197.0± 77.9$^{\#}$	174.1± 62.5$^{\#}$	190.7± 84.8$^{\#}$	176.5± 67.0$^{\#}$	<0.01
%TG-Extract	100	97.8± 12.6	121.3± 26.6$^{\#}$	156.8± 39.1$^{\#}$	176.2± 30.2$^{\#}$	185.2± 34.4$^{\#}$	173.2± 42.2$^{\#}$	171.2± 45.0$^{\#}$	162.4± 30.8$^{\#}$	156.5± 30.8$^{\#}$	145.7± 23.2$^{\#}$	159.3± 22.9$^{\#}$	0.24
$^{+}p_{P-E}$	-	0.63	0.42	0.68	0.46	0.25	0.10	0.11	0.11	0.29	0.08	0.41	0.23
%Total cholesterol-Placebo	100	99.3± 6.5	103.6± 9.1	102.6± 9.9	105.9± 8.9	104.7± 9.8	107.6± 12.5	100.9± 14.6	103.6± 9.6	99.5± 4.9	102.1± 12.6	102.6± 6.5	0.27
%Total cholesterol-Extract	100	99.4± 6.0	100.1± 5.2	101.0± 5.9	104.2± 8.9	97.9± 8.0	102.1± 9.8	97.4± 8.7	95.9± 7.3	96.7± 6.3	96.8± 5.7	102.7± 5.9	0.31
$^{+}p_{P-E}$	-	0.96	0.32	0.67	0.60	0.07	0.15	0.36	0.01	0.18	0.16	0.97	0.26
%HDL-c - Placebo	100	103.8± 9.9	100.2± 8.1	103.8± 11.1	97.5± 12.9	98.2± 13.7	96.0± 15.1	97.2± 13.9	96.1± 11.6	98.1± 10.9	99.9± 13.4	99.9± 12.4	<0.01
%HDL-c - Extract	100	101.4± 4.9	102.9± 7.9	99.4± 7.5	97.0± 8.2	94.6± 9.1	95.6± 8.0	91.7± 10.3$^{\#}$	92.3± 7.3$^{\#}$	95.5± 7.6	98.7± 6.3	101.7± 9.2	0.40
$^{+}p_{P-E}$	-	0.44	0.47	0.31	0.90	0.48	0.93	0.37	0.42	0.60	0.80	0.74	0.64
%LDL-c - Placebo	100	98.4± 8.7	101.6± 12.7	93.5± 12.4	98.2± 12.4	91.0± 14.5	97.0± 11.4	86.7± 17.9	93.3± 12.4	88.7± 9.9$^{\#}$	89.4± 18.2	93.5± 11.1	0.02
%LDL-c - Extract	100	98.8± 8.6	95.2± 8.3	94.7± 8.0$^{\#}$	98.4± 12.2	87.6± 11.7$^{\#}$	95.6± 10.2	89.9± 9.7$^{\#}$	88.3± 6.8$^{\#}$	88.3± 6.4$^{\#}$	89.4± 6.5$^{\#}$	95.3± 8.3	0.67
$^{+}p_{P-E}$	-	0.92	0.23	0.81	0.96	0.49	0.76	0.54	0.25	0.93	0.99	0.69	0.80

For normally distributed variables, data are presented as means ± SD. Repeated measures ANOVA was used for the comparisons (p_{time}, p_{trial}, $p_{time*trial}$). Paired samples t-test was used to compare each time point to the other intervention (p_{P-E}) and to baseline. TG; triglycerides, HDL-c; high-density lipoprotein cholesterol, LDL-c; low-density lipoprotein cholesterol. ^p trend from RMANOVA, $^{+}$p value from paired samples t-test, $^{\#}p \leq 0.05$ compared to baseline.

Supplementary Table S2: Percentage of change in basic biochemical markers in the two intervention groups for participants with BMI > 25kg/m^2.

	**-15**	**0**	**30**	**60**	**90**	**120**	**150**	**180**	**210**	**240**	**300**	**360**	$^{\wedge}p_{time}$ / $^{\wedge}p_{time*trial}$ / $^{\wedge}p_{trial}$
%Glucose -Placebo	100	106.5±16.1	122.5±27.1	93.9±15.0	102.8±11.3	108.5±10.9	104.8±8.4	102.7±14.0	99.9±5.8	101.3±6.1	97.7±7.1	95.0±8.1	0.57
%Glucose -Extract	100	110.2±10.8$^{\#}$	115.9±16.9$^{\#}$	99.7±10.3	103.0±18.0	102.9±10.5	101.7±8.6	100.9±6.8	101.2±7.2	94.7±6.2	92.3±6.1$^{\#}$	89.5±8.4$^{\#}$	0.85
$^{\dagger}p_{P-E}$	-	0.59	0.51	0.25	0.97	0.11	0.49	0.76	0.72	0.11	0.21	0.14	0.55
%Insulin-Placebo	100	-	698.4±342.5$^{\#}$	420.3±140.4$^{\#}$	368.4±173.2$^{\#}$	439.3±150.1$^{\#}$	369.1±64.5$^{\#}$	-	-	-	-	-	0.03
%Insulin-Extract	100	-	650.1±220.9$^{\#}$	616.4±449.2$^{\#}$	474.4±266.6$^{\#}$	435.1±215.4$^{\#}$	357.0±124.0$^{\#}$	-	-	-	-	-	0.40
$^{\dagger}p_{P-E}$	-	-	0.55	0.22	0.08	0.95	0.83	-	-	-	-	-	0.64
%TG-Placebo	100	93.4±8.8	120.9±17.3$^{\#}$	142.4±20.7$^{\#}$	147.4±23.3$^{\#}$	167.9±23.3$^{\#}$	158.6±18.6$^{\#}$	158.2±18.6$^{\#}$	150.2±24.4$^{\#}$	136.2±23.9$^{\#}$	132.7±12.4$^{\#}$	143.8±13.0$^{\#}$	0.16
%TG-Extract	100	97.0±6.7	118.0±6.9$^{\#}$	145.7±12.9$^{\#}$	168.2±38.1$^{\#}$	171.9±43.2$^{\#}$	181.0±34.4$^{\#}$	181.3±81.3$^{\#}$	170.0±60.9$^{\#}$	183.8±53.3$^{\#}$	122.5±12.5$^{\#}$	111.2±28.5	0.18
$^{\dagger}p_{P-E}$	-	0.36	0.66	0.60	0.31	0.85	0.25	0.55	0.51	0.07	0.19	0.12	0.42
%Total cholesterol-Placebo	100	98.3±8.1	99.2±2.4	97.3±6.6	106.7±4.3#	98.5±7.0	102.2±3.8	98.6±7.4	99.7±5.4	95.5±8.9	98.4±8.8	102.9±11.2	0.40
%Total cholesterol-Extract	100	99.5±2.4	102.3±4.5	97.2±4.7	102.2±4.7	99.4±3.8	102.4±5.1	97.5±5.6	97.7±6.0	98.3±7.8	96.8±5.4	99.2±4.2	0.54
$^{\dagger}p_{P-E}$	-	0.72	0.23	0.99	0.12	0.78	0.93	0.72	0.47	0.45	0.60	0.41	0.84
%HDL-c - Placebo	100	102.2±3.8	98.6±6.4	99.3±6.7	97.2±5.6	95.4±4.1$^{\#}$	93.7±4.8$^{\#}$	94.8±5.6$^{\#}$	94.6±8.4	96.9±5.3	99.5±5.2	99.8±6.6	0.76
%HDL-c - Extract	100	98.4±4.5	98.5±8.6	97.5±8.5	94.1±6.4$^{\#}$	94.7±8.4	93.3±8.2	92.4±10.7	91.8±10.0	92.7±13.6	92.0±7.9$^{\#}$	100.2±11.8	0.49
$^{\dagger}p_{P-E}$	-	0.21	0.97	0.68	0.42	0.84	0.89	0.45	0.57	0.44	0.08	0.94	0.50
%LDL-c - Placebo	100	96.0±14.6	96.6±3.1$^{\#}$	88.9±14.4$^{\#}$	107.9±8.7	92.1±11.8	99.9±5.1	95.6±16.1	97.4±12.6	92.4±18.4	95.8±17.5	101.9±18.7	0.24
%LDL-c - Extract	100	100.0±3.4	100.0±6.3	90.2±7.3$^{\#}$	96.5±5.2	91.1±5.9$^{\#}$	95.4±7.1	86.9±7.2$^{\#}$	89.7±6.3$^{\#}$	88.5±12.1$^{\#}$	95.6±9.3	98.2±7.6	0.37
$^{\dagger}p_{P-E}$	-	0.52	0.34	0.80	0.06	0.83	0.35	0.15	0.22	0.60	0.96	0.52	0.45

For normally distributed variables, data are presented as means ± SD. Repeated measures ANOVA was used for the comparisons (p_{time}, p_{trial}, $p_{time*trial}$). Paired samples t-test was used to compare each time point to the other intervention (p_{P-E}) and to baseline. TG; triglycerides, HDL-c; high-density lipoprotein cholesterol, LDL-c; low-density lipoprotein cholesterol. $^{\wedge}p$ trend from RMANOVA, $^{\dagger}p$ value from paired samples t-test, $^{\#}p \leq 0.05$ compared to baseline.

Supplementary Table S3: Percentage of change in oxidative and endogenous antioxidant biomarkers in the two intervention groups for participants with BMI < 25kg/m^2.

	**-15**	**0**	**30**	**60**	**90**	**120**	**150**	**180**	**210**	**240**	**300**	**360**	$^{\wedge}p_{time}$ / $^{\wedge}p_{time*trial}$ / $^{\wedge}p_{trial}$	$^{§†}p_{time}$
%UA-Placebo	100	103.8± 6.4	105.9± 6.1$^{\#}$	116.0± 13.6$^{\#}$	121.7± 14.5$^{\#}$	131.4± 28.8$^{\#}$	121.9± 27.1$^{\#}$	116.8± 29.7	107.0± 22.0	102.5± 15.2	103.4± 27.7	99.0± 13.6	<0.01	-
%UA-Extract	100	95.9± 4.2#	98.2± 3.6	111.7± 18.0$^{\#}$	116.5± 8.1$^{\#}$	113.4± 15.4$^{\#}$	108.2± 18.8	103.8± 15.2	101.7± 16.4	97.3± 10.8	91.4± 11.0$^{\#}$	94.7± 10.6	0.33	-
$^{†}p_{P-E}$	-	0.01	0.01	0.49	0.24	0.02	0.06	0.13	0.27	0.16	0.07	0.33	0.08	-
%TBARS-Placebo	100	-	-	96.1± 13.2	-	94.7± 11.6	-	91.2± 19.5	-	91.7± 23.8	79.6± 18.5$^{\#}$	77.3± 12.5$^{\#}$	<0.01	-
%TBARS-Extract	100	-	-	92.1± 9.4$^{\#}$	-	92.5± 11.0$^{\#}$	-	80.4± 10.6$^{\#}$	-	76.6± 15.0$^{\#}$	68.4± 15.2$^{\#}$	73.9± 16.5$^{\#}$	0.19	-
$^{†}p_{P-E}$	-	-	-	0.35	-	0.62	-	0.06	-	0.02	0.01	0.50	0.14	-
%PC-Placebo	100	98.2± 16.4	107.5± 21.2	105.5± 18.4	98.6± 15.0	96.2± 15.0	95.1± 13.2	96.3± 16.4	94.3± 17.0	101.8± 16.0	98.4± 24.5	114.6± 26.2	0.44	-
%PC-Extract	100	91.9± 6.0$^{\#}$	95.3± 11.9	94.0± 11.0	90.7± 10.1$^{\#}$	95.2± 9.6	100.4± 14.1	91.6± 11.2$^{\#}$	101.1± 20.6	95.2± 19.4	89.2± 10.4$^{\#}$	89.5± 11.9$^{\#}$	0.03	-
$^{†}p_{P-E}$	-	0.27	0.14	0.14	0.18	0.88	0.32	0.32	0.49	0.47	0.33	0.01	0.17	-
%SOD-Placebo	100	-	-	100.9 (79.9-119.2)	-	90.8 (76.4-164.7)	-	91.9 (70.9-120.4)	88.0 (55.9-92.8)	95.8 (76.9-113.7)	97.1 (65.0-115.4)	88.9 (62.4-100.5)	-	0.12
%SOD-Extract	100	-	-	85.2 (57.4-112.5)	-	72.6 (67.8-97.2)	-	87.7 (49.3-95.9)$^{\#}$	85.6 (55.6-107.9)	75.2 (58.0-132.4)	96.3 (72.6-114.1)	104.5 (67.8-119.6)	-	0.05
$^{†}p_{P-E}$	-	-	-	0.04	-	0.04	-	0.28	0.79	0.92	0.92	0.37	-	-
%GPx-Placebo	100	103.9± 4.1$^{\#}$	101.3± 2.9	100.3± 6.0	101.6± 8.6	99.5± 7.7	102.5± 10.4	99.2± 6.1	102.1± 10.4	99.1± 10.0	101.6± 7.7	104.6± 4.9$^{\#}$	0.25	-
%GPx-Extract	100	104.9± 5.7$^{\#}$	103.3± 3.6$^{\#}$	101.3± 6.8	101.7± 8.7	101.3± 6.9	103.6± 10.5	101.1± 10.5	99.3± 7.9	101.2± 8.1	101.2± 6.1	102.4± 10.4	0.83	-
$^{†}p_{P-E}$	-	0.65	0.19	0.62	0.99	0.64	0.81	0.51	0.59	0.54	0.89	0.57	0.83	-

For normally distributed variables, data are presented as means ± SD. Repeated measures ANOVA was used for the comparisons (p_{time}, p_{trial}, $p_{time*trial}$). Paired samples t-test was used to compare each time point to the other intervention (p_{P-E}) and to baseline. For skewed variables, data are presented as the median (25th-75th quartile). Friedman's 2-way ANOVA by ranks was used for the estimation of the time effect in each intervention group ($^{§}p_{time}$) and Wilcoxon paired test for differences compared to baseline. Trial-related pairwise comparisons were performed using Wilcoxon paired test (p_{P-E}). UA; uric acid, TBARS; thiobarbituric acid reactive substances, PC; protein carbonyls, SOD; superoxide dismutase, GPx; glutathione peroxidase. $^{\wedge}p$ trend from RMANOVA, $^{†}p$ value from Paired samples t-test, Friedman, Wilcoxon test, $^{\#}p \leq 0.05$ compared to baseline

Supplementary Table S4: Percentage of change in oxidative and endogenous antioxidant biomarkers in the two intervention groups for participants with BMI > 25kg/m^2.

	**-15**	**0**	**30**	**60**	**90**	**120**	**150**	**180**	**210**	**240**	**300**	**360**	$^{\wedge}p_{time}$ / $^{\wedge}p_{time*trial}$ / $^{\wedge}p_{trial}$	$^{§†}p_{time}$
%UA-Placebo	100	100.1± 5.2	103.5± 9.7	107.9± 10.8	112.2± 6.6$^{\#}$	109.1± 8.0$^{\#}$	102.9± 4.5	96.5± 6.9	95.5± 9.2	91.5± 6.9$^{\#}$	89.9± 13.3	97.4± 19.2	0.29	-
%UA-Extract	100	105.7± 8.3	109.3± 5.1$^{\#}$	109.9± 4.8$^{\#}$	117.9± 13.8$^{\#}$	121.7± 10.0$^{\#}$	116.8± 10.4$^{\#}$	117.3± 24.3	108.2± 18.2	115.2± 15.6$^{\#}$	94.6± 6.1$^{\#}$	91.6± 10.8	0.04	-
$^{†}p_{P-E}$	-	0.19	0.17	0.55	0.14	0.02	0.01	0.07	0.15	0.00	0.25	0.30	0.03	-
%TBARS-Placebo	100	-	-	100.4± 16.2	-	97.5± 17.3	-	92.5± 16.9	-	91.1± 30.5	86.1± 22.7	79.2± 15.5$^{\#}$	0.20	-
%TBARS-Extract	100	-	-	107.1± 23.9	-	96.4± 10.6	-	90.3± 11.2	-	83.7± 18.1$^{\#}$	83.6± 9.0$^{\#}$	83.0± 18.7$^{\#}$	0.70	-
$^{†}p_{P-E}$	-	-	-	0.39	-	0.85	-	0.74	-	0.63	0.79	0.72	0.96	-
%PC-Placebo	100	97.8± 9.2	111.4± 21.6	101.0± 9.6	107.1± 16.2	116.5± 23.0	100.1± 12.1	103.2± 9.4	111.8± 23.1	107.6± 14.7	107.5± 12.0	103.9± 17.8	0.64	-
%PC-Extract	100	88.5± 7.8$^{\#}$	88.8± 15.9	93.9± 16.5	86.6± 10.6$^{\#}$	85.5± 15.0$^{\#}$	86.8± 20.6	91.4± 14.5	93.7± 21.4	88.6± 10.5$^{\#}$	89.3± 14.0	84.6± 20.8	0.26	-
$^{†}p_{P-E}$	-	0.11	0.10	0.42	0.07	0.02	0.15	0.19	0.08	<0.01	0.01	0.07	<0.01	-
%SOD-Placebo	100	-	-	104.5 (81.3-119.2)	-	102.5 (77.5-149.2)	-	80.2 (60.0-127.6)	100.1 (85.4-120.2)	89.4 (80.5-107.8)	86.9 (78.8-105.5)	89.9 (63.7-114.2)	-	0.78
%SOD-Extract	100	-	-	93.3 (84.2-98.1)	-	94.8 (73.5-97.9)	-	95.2 (79.2-106.6)	90.4 (80.0-100.6)	87.1 (78.9-93.0)	80.7 (68.5-89.3)$^{\#}$	92.6 (70.6-107.1)	-	0.16
$^{†}p_{P-E}$	-	-	-	0.49	-	0.17	-	0.17	0.12	0.23	0.12	0.39	-	-
%GPx-Placebo	100	103.4± 4.0	95.6± 5.2	98.6± 3.8	97.9± 6.3	95.1± 3.9$^{\#}$	98.6± 6.5	98.8± 5.3	97.1± 9.1	99.7± 5.6	100.4± 6.2	99.3± 5.9	0.28	-
%GPx-Extract	100	104.1± 5.7	99.3± 5.8	97.8± 4.9	96.5± 7.1	95.7± 7.2	101.0± 4.7	100.7± 11.1	98.1± 4.4	100.5± 6.6	102.5± 7.7	105.7± 7.5	0.69	-
$^{†}p_{P-E}$	-	0.83	0.36	0.55	0.77	0.87	0.42	0.66	0.68	0.72	0.65	0.16	0.48	-

For normally distributed variables, data are presented as means ± SD. Repeated measures ANOVA was used for the comparisons (p_{time}, p_{trial}, $p_{time*trial}$). Paired samples t-test was used to compare each time point to the other intervention (p_{P-E}) and to baseline. For skewed variables, data are presented as the median (25th-75th quartile). Friedman's 2-way ANOVA by ranks was used for the estimation of the time effect in each intervention group ($^{§}p_{time}$) and Wilcoxon paired test for differences compared to baseline. Trial-related pairwise comparisons were performed using Wilcoxon paired test ($^{§}p_{time}$). UA; uric acid, TBARS; thiobarbituric acid reactive substances, PC; protein carbonyls, SOD; superoxide dismutase, GPx; glutathione peroxidase, $^{\wedge}p$ trend from RMANOVA, $^{†}p$ value from Paired samples t-test, Friedman, Wilcoxon test, $^{\#}p \leq 0.05$ compared to baseline
